# B lymphocytes transdifferentiate into immunosuppressive erythroblast-like cells

**DOI:** 10.3389/fimmu.2023.1202943

**Published:** 2023-07-21

**Authors:** Zhe Yang, Zheng Wang, Lei Wu, Ying Wang, Zhihui Xu, Ying Liu, Fangfang Wang, Duonan Yu

**Affiliations:** ^1^ Institute of Translational Medicine, Yangzhou University Medical College, Yangzhou, China; ^2^ Jiangsu Key Laboratory of Experimental and Translational Non-coding RNA Research, Yangzhou University, Yangzhou, China; ^3^ Pathology Department, Affiliated Hospital of Yangzhou University, Yangzhou University, Yangzhou, China; ^4^ College of Life Sciences, Nanjing Agricultural University, Nanjing, China; ^5^ Department of Hematology, Yangzhou University Clinical Medical College, Yangzhou, China

**Keywords:** CD45^+^ EPC, TER cells, immunosuppressive erythroblast, B lymphocyte, ROS

## Abstract

Recent studies have demonstrated that a particular group of nucleated cells that exhibit erythroid markers (TER119 in mice and CD235a in humans) possess the ability to suppress the immune system and promote tumor growth. These cells are known as CD45^+^ erythroid progenitor cells (EPCs). According to our study, it appears that a subset of these CD45^+^ EPCs originate from B lymphocytes. Under conditions of hypoxia, mouse B lymphoma cells are capable of converting to erythroblast-like cells, which display phenotypes of CD45^+^TER119^+^ cells, including immunosuppressive effects on CD8 T cells. Furthermore, non-neoplastic B cells have similar differentiation abilities and exert the same immunosuppressive effect under anemia or tumor conditions in mice. Similar B cells exist in neonatal mice, which provides an explanation for the potential origin of immunosuppressive erythroid cells in newborns. Additionally, CD19^+^CD235a^+^ double-positive cells can be identified in the peripheral blood of patients with chronic lymphocytic leukemia. These findings indicate that some CD45^+^ EPCs are transdifferentiated from a selective population of CD19^+^ B lymphocytes in response to environmental stresses, highlighting the plasticity of B lymphocytes. We anticipate a potential therapeutic implication, in that targeting a specific set of B cells instead of erythroid cells should be expected to restore adaptive immunity and delay cancer progression.

## Introduction

A vast range of somatic cells are capable of being re-programmed. Fibroblasts, for example, can be transformed into pluripotent stem cells (1–3), while myeloid and lymphoid cells can be re-educated to become myeloid-derived suppressor cells (MDSCs) (4–8), regulatory B cells (Bregs) (9–11), and regulatory T cells (Tregs) (12–14). C/EBPα regulates the differentiation of B cells toward macrophages (15–18), and Hoxb5 transdifferentiates B cell progenitor cells (pro/pre-B) into T cells (19). B-cell lymphoma and myeloid cells can switch their identities (20). The differentiation of B lymphoma cells into histiocytic tumor cells and CD19^+^ pro-B cells into dendritic cells has also been observed (21). Compared to fibroblasts, which requires artificial modification to achieve transdifferentiation, B cells appear to have higher differentiation plasticity and spontaneous transdifferentiation appears to be possible.

Immunosuppressive erythroid cells are abundant in the spleens of neonatal mice and the umbilical cord blood of humans (22, 23). In addition to newborns, these CD71^+^ erythroid cells, with an immunosuppressive function, also exist in adults (24). In advanced tumors, cell types that exhibit an immunosuppressive phenotype (CD45^+^CD71^+^TER119^+^) and cells that exert regulatory effects on distal tumors (CD45^-^CD71^+^TER119^+^) have also been discovered (25, 26). These unconventional erythroid cells, which are identified by the presence of the erythroid marker TER119 in mice or CD235a in humans, have also been linked to the development of immune deficiency (27), feto-maternal tolerance, and infectious diseases (28). However, there has been no report showing that these erythroid cells with immune regulatory functions are derived from hematopoietic stem cells. Therefore, the origin of these immunosuppressive erythroid cells remains unknown.

Through induction of hypoxia, it is possible for non-erythroid cells, such as fibroblasts and cancer cells, to express the proteins associated with red blood cells (RBCs) (29, 30). This finding indicates that the RBC-related markers (especially TER119 or CD235a) are likely to appear on non-erythroid cells under certain physiological or pathological conditions. Here, we demonstrate that cells identified as CD45^+^ EPCs may not consist exclusively of erythroblasts, but rather that some may be cells that have undergone differentiation from B lymphocytes.

## Materials and methods

### Cell lines and cell culture

A murine P53ER1 (ER1) cell line was established by culturing B lymphoma cells (31) on a monolayer of murine bone marrow-derived and mitomycin-treated S17 stromal cells (32). Cells were maintained in RPMI 1640 supplemented with 10% fetal bovine serum (FBS, HyClone) and 100 μM interleukin-7 (IL7, R&D Systems, Cat. No. 407-ML) at 37°C in a humidified incubator with 5% CO_2_. A short-term culture of primary B lymphocytes from mouse spleen and bone marrow was also maintained on an S17 feeder layer in RPMI 1640 medium with 10% FBS and IL7. Madin-Darby canine kidney (MDCK) and African green monkey kidney (Vero E6) cells were cultured in DMEM with 10% FBS. B16F10 melanoma cells (B16F10) and Lewis lung carcinoma (LLC) cells were obtained from the American Type Culture Collection (ATCC) and cultured in DMEM/H (HyClone) containing 10% FBS and 1% penicillin/streptomycin (Beyotime Biotechnology). Cell lines were routinely validated and were free of mycoplasma contamination.

### Flow cytometry and antibodies

Antibody staining was performed in PBS containing 2% BSA or FBS. Data were collected using a FACSCanto system (BD Bioscience) and analyzed with FlowJo software (TreeStar). The following fluorescence-conjugated antibodies were used: anti-B220 (BD Biosciences, Cat. No. 553092), anti-CD19 (Biolegend, Cat. No. 115506), anti-CD45 (Biolegend, Cat. No. 103108), anti-TER119 (BD Biosciences, Cat. No. 557909), anti-TER119 (Thermo Fisher, Cat. No. MA5-17821), anti-CD71 (BD Biosciences, Cat. No. 553267), anti-CD8 (Biolegend, Cat. No. 10072), anti-CD44 (eBioscience, REF. 12-0441-83), anti-PD-1 (RMP1-30) (eBioscience, REF. 11-9981-81), anti-CD45.1 (Thermo, Cat. No. 12-0453-82), anti-CD45.2 (BD Pharmingen, Cat. No. 560696), anti-CD235a (BD Pharmingen, Cat. No. 551336), anti-CD45 (BD Pharmingen, Cat. No. 555483), and anti-CD19 (BD Pharmingen, Cat. No. 555412). ROS production was measured by labeling cells with 2’ and 7’-dichlorofluorescin diacetates (H2DCFDA, MedChemExpress, Cat. No. HY-D0940). Mouse CD3 epsilon antibody (R&D, Cat. No. MAB484-SP) and mouse CD28 antibody (R&D, Cat. No. MAB4832-SP) were used to stimulate CD8^+^ cell proliferation *in vitro*.

### Polymerase chain reaction

Variable (V), diversity (D) and joining (J) (VDJ) rearrangement of mouse B lymphocytes was measured by genomic DNA PCR under the following conditions, as previously published (33): denaturation at 95°C for 30 seconds, annealing at 62°C for 45 seconds, and extension at 72°C for 60 seconds. All reactions were carried out for 35 cycles with 5-minute initial denaturing and 10-minute final extension. PCR analysis for gag-myc sequence was also performed using genomic DNA. Primers used for human VDJ rearrangement were FR3A, LJH, and VLJH, as previously described (33). For nested PCR, primers FR3A and LJH were used for the first round of PCR, and FR3A and VLJH for the second round. Reactions were pre-denatured at 94°C for 6 min, followed by 40 cycles of denaturation at 94°C for 1 min, annealing at 55°C for 1 min, and extension at 72°C for 1 min, with final extension at 72°C for 10 min. All primers used for DNA PCR are listed in **Supplementary Table 1**.

### Quantitative real time PCR

Total RNA was extracted with TRIzol (Invitrogen, Cat. No. 15596026) and 1 μg total RNA was used for first-strand cDNA synthesis. qRT-PCR was performed on a 7500 Fast Real-Time PCR System (Applied Biosystems). Primers used for PCR analysis are listed in **Supplementary Table 1**.

### Western blot

Whole-cell protein lysates were prepared using lysis buffer with 1:500 protease inhibitor mixture (Sigma-Aldrich). Proteins were separated on 10% SDS-PAGE gel, transferred to polyvinylidene fluoride membranes, and blotted with the primary antibodies followed by secondary antibodies. Protein blots were visualized using SuperSignal West Femto Maximum Sensitivity Substrate (Thermo, Cat. No. 34095) as directed by the manufacturer. Anti-artemin antibody and anti-HIF1a antibody were purchased from Abcam (Cat. No. ab178434) and Absin (Cat. No. abs130612), respectively. Anti-β-actin monoclonal antibody (Absin, Cat. No. abs149632) was used as an internal control.

### Immunochemical staining

Sorted CD45^+^CD19^+^TER119^+^ cells were immunocytochemically stained using rat anti-mouse TER119 antibody (BD Biosciences, Cat. No. 557909) followed by labeling with goat anti-rat IgG (H+L). Cells were counterstained with hematoxylin. For immunofluorescence staining, the sorted cells were cytospun onto glass slides, fixed with 4% paraformaldehyde, and blocked with PBS containing 3% FBS and 0.5% Tween 20. Primary antibodies were incubated at room temperature for 1 h. DAPI was used to stain nuclei. Cells were observed under a confocal microscope (Leica, TCS SP8 STED).

### Fluorescence *in situ* hybridization

A Y chromosome marker gene (Sry) was probed using Cy3-labeled Sry probes (RiboBio™, Fluorescence *In Situ* Hybridization Kit, Cat. No. C10910). Cells on slides were denatured in 70% formamide/2× SSC (1× SSC is 0.15M NaCl/0.015M sodium citrate, pH 7) at 70°C for 3 min and dehydrated in ethanol. Subsequently, 10 μL hybridization mixture containing 40 ng probes and 0.5 μg yeast genomic DNA (as blocking) was denatured for 5 min at 70°C and reannealed for 45–60 min at 42°C. Hybridization was carried out overnight at 42°C in a moist chamber. Samples were observed via confocal microscopy (Leica, TCS SP8 STED).

### Cell isolation and sorting

Micro-magnetic beads for sorting of mouse and human cells were purchased from Miltenyi Biotec. Cell sorting was performed according to the manufacturer’s instructions. The micro-magnetic beads used included mouse CD8 (TIL) (Cat. No. 130-116-478), mouse CD19 (Cat. No. 130-121-301), mouse Ter-119 (Cat. No. 130-049-901), and human CD19 (Cat. No.130-050-301) MicroBeads. LS separation columns were also purchased from Miltenyi (Cat. No. 130-042-401). To enrich CD8^+^ T cells, C57BL6 mice were stimulated with LCMV-Cl13 (2×10^6^ PFU per mouse) and CD8^+^ T cells from spleens were purified using CD8a T cell magnetic beads (Miltenyi Biotec) or a FACSAriaII sorter (BD Biosciences). Briefly, mice were infected with LCMV-Cl13 virus and CD8^+^ cells were isolated using a CD8a^+^ T cell isolation kit according to the manufacturer’s instructions. Purified T cells were further stained with CD44, PD-1, and CD45.2 antibodies and sorted using a flow cytometer to obtain CD8^+^CD44^hi^PD-1^hi^CD45.2^+^ T cells for *in vivo* and *ex vivo* killing assays. To isolate peripheral blood mononuclear cells (PBMCs), 60% Percoll (Absin, Cat. No. abs9417) working solution with an equal volume of blood was mixed and centrifuged at 800g for 30 min. The middle layer of cells containing PBMCs was carefully collected and washed with PBS.

### *In vitro* induction of TER cells from CD19^+^ B lymphocytes

CD19^+^ B lymphocytes derived from mouse bone marrow were cultured in medium containing CoCl_2_ (100 μM) and IL6 (200 ng/ml) (Absin, Cat. No. abs04084). Cells were analyzed via flow cytometry.

### Animal models

Mice with severe combined immunodeficiency (SCID) used in the *in vivo* T cell killing effect assay were housed in SPF flow cabinets. To induce acute anemia, C57BL6/J mice (CD45.2) were injected intraperitoneally (i.p.) with 5FU (MedChemExpress, Cat. No. HY-90006) at a dose of 70 mg/kg mouse weight. To establish xenograft tumor models, 1×10^6^ LLCs or 5×10^5^ B16F10 cells were resuspended in 150 μl PBS and subcutaneously injected into the right flank of C57BL/6 mice. Tumor-bearing mice were infected with pathogens at different time points after tumor cell injection. The acute hemolytic anemia model was constructed by intraperitoneal injection of phenylhydrazine (PHZ) (Merck, Cat. No. 114715-5G) at a dose of 50 mg/kg. SJL mice (CD45.1) were used to sort CD19^+^ B cells for adoptive transplantation. All animal experiments were performed using protocols approved by the Animal Care and Use Committee of the Yangzhou University Medical College.

### RNA sequencing

Total RNAs from ER1 (CD45^+^TER119^+^CD19^-^, hereafter BL1-TER119), ER1 (CD45^+^TER119^-^CD19^-^), and ER1 (CD45^+^TER119^-^CD19^+^, hereafter BL1-CD19) cells were extracted using TRIzol reagent. The RNA-Seq library was constructed according to the strand-specific RNA sequencing library preparation protocol and sequenced on an Illumina HiSeq 2000. The full RNA-Seq datasets were uploaded to the Gene Expression Omnibus (GEO) database (accession code: GSE201915). Total RNAs from CD45^+^ TER cells in neonatal mouse spleens, erythroblasts from 14.5-day fetal livers, and CD19^+^ B lymphocytes from adult mouse spleens were extracted for RNA-Seq. These datasets were also deposited in the GEO database under accession number GSE201937. The differentially expressed genes were matched to Gene Set Enrichment Analysis (GSEA)-Gene Oncology (GO) datasets (34). The GO terms with adjusted *p* < 0.05 were regarded as significantly enriched.

### Virus titration

The LCMV-Armstrong clone 13 (Cl13) strain was provided by Dr. Ye at the Third Military Medical University of China. For quantification of LCMV-Cl13 loads, the lysate volume of LCMV-Cl13-infected Vero cells was measured. Total RNA was extracted from the lysate and subjected to reverse transcription using the PrimeScript RT Master Mix kit (Takara, Cat. No. RR036A). LCMV-specific primer (GP-R: GCAACTGCTGTGTTCCCGAAAC) was used for first-strand cDNA synthesis. qRT-PCR with LCMV glycoprotein-specific primers (GP-F: CATTCACCTGGACTTTGTCAGACTC and GP-R: GCAACTGCTGTGTTCCCGAAAC) were used to evaluate viral loads in Vero cells. The Cq (quantification cycle) value for LCMV-Armstrong RNA that had been previously titrated by plaque assay was used to generate a standard curve. The PFU of LCMV-Cl13 in Vero cell lysates was calculated according to the following formula: lg (PFU) = slop*Cq + y-intercept; and the titration was calculated as PFU/ml = lg (PFU)/lysate volume.

### Animal adoptive transplantation assay

CD45.1 B lymphocytes from male SJL mouse bone marrow were isolated using CD19 MicroBeads (Miltenyi Biotec, Cat. No. 130-121-301) and were injected into CD45.2 mice (C57BL6/J) through the retro-orbital sinus. Five days after transplantation, transdifferentiated cells were examined via flow cytometry.

### *In vivo* killing assay

The *in vivo* killing assay was performed as previously described (35, 36). Briefly, the spleen mononuclear cells encapsulated by LCMV gp33 (Absin, Cat. No. abs45152613) were used as target cells. The gp33-coated splenic cells were stained with 2.5 μM carboxyfluorescein succinimidyl ester (CFSE, MedChemExpress, Cat. No. HY-D0938), and the uncoated spleen mononuclear cells were stained with 0.25 μM CFSE as a control. TER cells (BL1-TER119 cells or CD45^+^CD19^+^TER119^+^ cells) and their control cells (BL1-CD19 cells or CD19^+^ B lymphocytes) were mixed with LCMV-Cl13-stimulated CD8^+^CD44^hi^PD-1^hi^ T cells and LCMV gp33-coated target cells, and these were co-injected intravenously into male SCID mice or C57BL6 mice. After 24 hours, the recipient mice were euthanized and the spleens removed for examination of the lysis effect of CD8^+^ T cells on target cells. Killing percentage (%) was calculated using following formula: 100 - [100 × (% CFSE^high^ immunized mouse/% CFSE^low^ immunized mouse)/(% CFSE^high^ naive mouse/% CFSE^low^ naive mouse). The CFSE^high^ cells were spleen mononuclear cells stained with a high concentration of CFSE (2.5 μM) and the CFSE^low^ cells were those stained with low concentration of CFSE (0.25 μM). Unless otherwise specified, the ratio of test cells (TER cells) to CD8^+^ T cells was 2:1.

### *Ex vivo* killing assay

The *ex vivo* killing assay was performed as previously described (35, 36). Peptide-pulsed target cells were mixed with sorted CD8^+^ T cells at a ratio of 2:1 and co-cultured for 6 hours. CFSE staining was conducted in a similar manner as for *in vivo* assay. Killing percentage (%) = 100 - [100 × (% CFSE^high^ immunized mouse/% CFSE^low^ immunized mouse)/(% CFSE^high^ naive mouse/% CFSE^low^ naive mouse)]. The CFSE^high^ cells were stained with 2.5 μM CFSE and the CFSE^low^ cells were stained with a low concentration of CFSE (0.25 μM).

### T cell proliferation assay

The T cell proliferation assay was performed as previously described (37). First, 24-well plates were coated with monoclonal antibody against CD3. T cell proliferation was analyzed by labeling T cells with CFSE at a concentration of 5 μM according to the manufacturer’s instructions. Briefly, cells were resuspended in warm PBS containing 5 μM CFSE and incubated at 37°C for 10 min. CFSE-labeled mouse or human PBMCs were added to the plates along with test cells and anti-CD3 antibody for co-stimulation. In ROS elimination experiments, the ER1 (TER119^+^) cells and the CD45^+^CD19^+^TER119^+^ TER cells were treated with 300 μM apocynin (49-hydroxy-39-methoxyacetophenone, MedChemExpress, Cat. No. HY-N0088) for 30 min in order to inhibit the activity of NADPH oxidase. For all experiments, cells were analyzed via flow cytometry 72 h after culturing. The anti-CD3 and anti-CD28 antibodies were used to co-stimulate CD8^+^ T cells.

### Human sample collection and processing

Peripheral blood was collected from cancer patients. Experiments were approved by the Ethic Committees of the Institutional Review Board of the Jiangsu Subei People’s Hospital. Written statements of informed consent were provided by all the participants before sample collection. Mononuclear cells were freshly isolated after lysis of RBCs.

### Statistical analysis

Statistical analysis was conducted using Prism 7.0 (GraphPad). Results are reported in the form mean ± standard deviation (SD). Two-tailed Student’s t-tests were conducted to calculate P values. In all experiments, no data points or animals were excluded from statistical analysis. In comparisons, *p* < 0.05 was considered to indicate statistically different values and *p* < 0.01 to indicate significantly different values.

## Results

### Mouse B lymphoma cells transdifferentiate into CD45^+^ erythroblast-like cells

**Figure 1:**
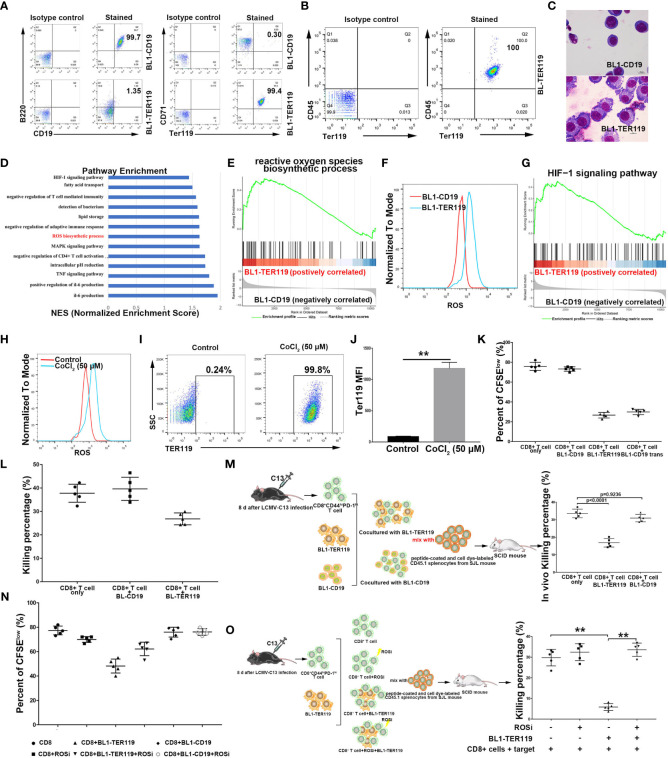
Mouse B lymphoma cells transdifferentiate into CD45^+^ TER cells. **(A)** Flow cytometry showing B cell lineage markers CD19/B220 and erythroid differentiation markers TER119/CD71 on BL1-CD19 and BL1-TER119 cells. Anti-B220-APC, anti-CD19-PE, anti-TER119-APC, and anti-CD71-PE antibodies were used. **(B)** Flow cytometric analysis of CD45 and TER119 on BL1-TER119 cells. Anti-CD45-FITC and anti-TER119-APC antibodies were used. **(C)** Morphological observation (1000×) of BL1-CD19 cells and BL1-TER119 cells stained with Wright-Giemsa. **(D)** Pathway enrichment analysis performed via gene set enrichment analysis (GSEA). Significantly enriched items (nominal *P*<0.05) in BL1-TER119 cells compared with BL1-CD19 cells. **(E)** Enrichment plot of the ROS biosynthetic process: comparison between BL1-TER119 cells and BL1-CD19 cells. **(F)** Intracellular ROS production in BL1-TER119 and BL1-CD19 cells as quantified by flow cytometry. **(G)** Enrichment plot of the HIF-1 signaling pathway: comparison between BL1-TER119 cells and BL1-CD19 cells. **(H)** ROS production in CoCl_2_-induced and -uninduced BL1-CD19 cells as quantified by flow cytometry. **(I)** Flow cytometric analysis of TER119 in CoCl_2_-induced BL1-CD19 and CoCl_2_-uninduced BL1-CD19 cells. BL1-CD19 cells were stressed by CoCl_2_ at a concentration of 50 μM for 5 days. Uninduced BL1-CD19 cells were used as a negative control. **(J)** Quantitative analysis of TER119 on CoCl_2_-induced and -uninduced BL1-CD19 cells under flow cytometry (n=5). **(K)** The proliferative capacity of CFSE-labeled CD8^+^ T cells in response to anti-CD3 and anti-CD28, analyzed after co-culture with BL1-CD19, BL1-TER119, or BL1-CD19^trans^ (CoCl_2_-induced BL1-CD19) cells. **(L)** The inhibitory effect of BL1-TER119 compared to BL1-CD19 cells on CD8^+^ T cell killing efficiency, detected *in vitro* by co-culturing the BL1 cells with sorted CD8^+^ T cells (sorted from LCMV-C13-infected C57BL6 mice) and evaluating the *ex vivo* T cell killing efficiency after 6 hours (n=5). C57BL6 mouse splenocytes coated with GP33 were used as target cells. **(M)** The inhibitory effect of BL1-TER119 compared to BL1-CD19 cells on CD8+ T cell killing efficiency, detected *in vivo* by co-injecting BL1 cells with sorted CD8^+^ T cells (from LCMV-C13-infected C57BL6 mice) and evaluating the *in vivo* T cell killing efficiency after 24 hours (n=5). **(N)** The inhibitory effect of BL1-TER119 compared to BL1-CD19 cells on CD8^+^ T cell proliferation, analyzed *in vitro* by co-culturing CFSE-labeled CD8^+^ T cells with BL1 cells at a ratio of 2:1 (BL1:CD8^+^ T) (n=5). CD8^+^ T cell proliferation was then evaluated in the presence or absence of APO (ROSi), an NADPH oxidase inhibitor that blocks ROS production, at a concentration of 300 nM. **(O)**. Schematic diagram and the results of an *in vivo* assay quantifying the effect of ROS inhibitor (ROSi) on the immunosuppressive function of BL1-TER119 cells. The *in vivo* killing assay quantifies the ability of CD8^+^ T cells isolated from the LCMV-13-stimulated mice to kill GP33-pulsed splenocytes *in vivo*. n=5 mice per group; **P<0.01 (t-test).

We have previously demonstrated transdifferentiation between B lymphoid cells and myeloid cells (20). Upon observing a Myc-induced and p53-inducible B lymphoma cell line BL1 that we established (**Supplementary Figure 1A**), we noted that the cells that were initially in suspension spontaneously adhered to the culture dish as the cell density increased. Flow cytometry analysis revealed that the adherent cells lacked B-cell marker CD19, while a proportion of these cells exhibited expression of TER119, an erythroid cell-specific marker. After sorting, the cells that expressed TER119 were given the name BL1-TER119 while the B lymphoma cells that were still CD19-positive were named BL1-CD19 (**Figure 1A**). BL1-TER119 cells exhibited comparable surface marker traits to CD45^+^ EPCs (CD45^+^TER119^+^) (**Figure 1B**; **Supplementary Figure 1B**). Compared to the original BL1-CD19 cells, BL1-TER119 cells are larger and contain more cytoplasm (**Figure 1C**). Additionally, these cells still retain the gag-myc and VJ558-JH4 rearrangement (**Supplementary Figure 1C**), confirming their B-lymphoid tumor origin. To examine the difference between BL1-TER119 cells and the original BL1-CD19 cells, we performed transcriptome sequencing. Gene set enrichment analysis (GSEA) suggested that BL1-TER119 cells acquire distinct immunological characteristics compared to BL1-CD19 cells (**Supplementary Figure 1D**). For instance, the mRNAs of two immunosuppressive genes, NOX2 (38) and Arg2 (39, 40), were highly expressed in BL1-TER119 cells compared to BL1-CD19 cells (**Supplementary Figure 1E**).

Immunosuppressive CD45^+^ EPCs share a common trait with other immunosuppressive myeloid cells: the production of excessive reactive oxygen species (ROS). This was confirmed by our RNA-sequencing data, which revealed significant enrichment of ROS biosynthetic processes in BL1-TER119 compared to BL1-CD19 (**Figures 1D–F**). Hydrogen peroxide (H_2_O_2_) elevated ROS levels and effectively stimulated production of hemoglobin in BL1-TER119 cells (**Supplementary Figure 1F**). Furthermore, hypoxia-inducible factor 1 (HIF-1) signaling was enriched in BL1-TER119 cells (**Figure 1G**), suggesting that hypoxia may be a factor that leads to transdifferentiation. To test this hypothesis, BL1-CD19 cells were treated with cobalt chloride (CoCl_2_), a compound that induces hypoxia (41). CoCl_2_ treatment resulted in high expression of HIF1α protein (**Supplementary Figure 1G**), higher intracellular ROS levels, and a surprising TER119 induction in BL1-CD19 cells (**Figures 1H–J**; **Supplementary Figure 1H**). CoCl_2_ treatment of BL1-CD19 cells (BL1-CD19 ^trans^) led to a much lower rate of proliferation of CD8^+^ T cells in a co-culture system, and a similar phenotype of BL1-TER119 cells (**Figure 1K**). In contrast, treatment of BL1-TER119 cells with Lw6, an HIF1α inhibitor, for 48 hours significantly decreased TER119 expression, with a reduced ROS level in cells (**Supplementary Figures 1I–K**, **2A-C**).

To assess the effect of BL1-TER119 cells on T cell functions, we infected C57/BL6 mice with lymphocytic choriomeningitis virus clone 13 (LCMV-13) to enrich CD8^+^ T lymphocytes. Co-culture experiments indicated that BL1-TER119 cells possessed the ability to impede the efficacy of CD8^+^ T cells (**Figure 1L**). *In vivo* killing analysis demonstrated a substantial reduction of peptide-coated splenocytes in the BL1-TER119 group, whereas BL1-CD19 cells failed to inhibit the killing of peptide-coated splenocytes by T cells (**Figure 1M**). To further confirm the role of ROS in the transition between BL1-CD19 and BL1-TER119 cells, BL1-TER119 cells were treated with the ROS inhibitor (ROSi) apocynin (APO). The inclusion of APO successfully mitigated the suppressive impact of BL1-TER119 cells on the proliferation of CD8^+^ T cells (**Figure 1N**; **Supplementary Figure 2D**). *In vivo* T cell killing experiments verified that ROS serves as the primary factor in suppression of the immune response for BL1-TER119 cells (**Figure 1O**). Interestingly, PCR analysis demonstrated that BL1-TER119 cells acquired TER119 expression but failed to express erythroid genes, including Hbb-b1, Ank1, Gypa, Alas2, Epb4.9, EpoR, and Gata1 (**Supplementary Figure 2E**), indicating that these cells are not necessarily erythroid cells. Additionally, Myc5, a mouse B lymphoma-derived cell line that we established, also expressed TER119, while murine B lymphoma cell lines 38B9 and A20 exhibited absence of TER119 (**Supplementary Figure 2F**). These results indicate that the induction of immunosuppressive TER cell-like cells from mouse B lymphoma cells is strongly influenced by ROS or affected by stimulation with hypoxic conditions. Taken together, these findings provide evidence that CD19^+^/B220^+^ mouse B lymphoma cells can undergo spontaneous differentiation into immunosuppressive CD45^+^ EPC-like cells.

### Induction of non-cancerous murine B lymphocytes into CD45^+^ erythroblast-like cells

To explore whether non-cancerous B lymphocytes possess the ability to transdifferentiate into CD45^+^ EPC-like cells, we conducted a bioinformatics analysis of the documented sequencing data from CD45^+^ EPCs (accession code: GSE106384), which have been found to possess immunosuppressive capabilities (25), and CD45-negative TER cells (CD45^-^TER) (accession code: GSE109429), which govern the metastasis of tumors (26). Transcriptome analysis indicated that CD45^+^ EPCs and CD45^-^TER cells exhibited a strong tendency to be B lymphocyte-derived (**Supplementary Figures 3–5**).

**Figure 2:**
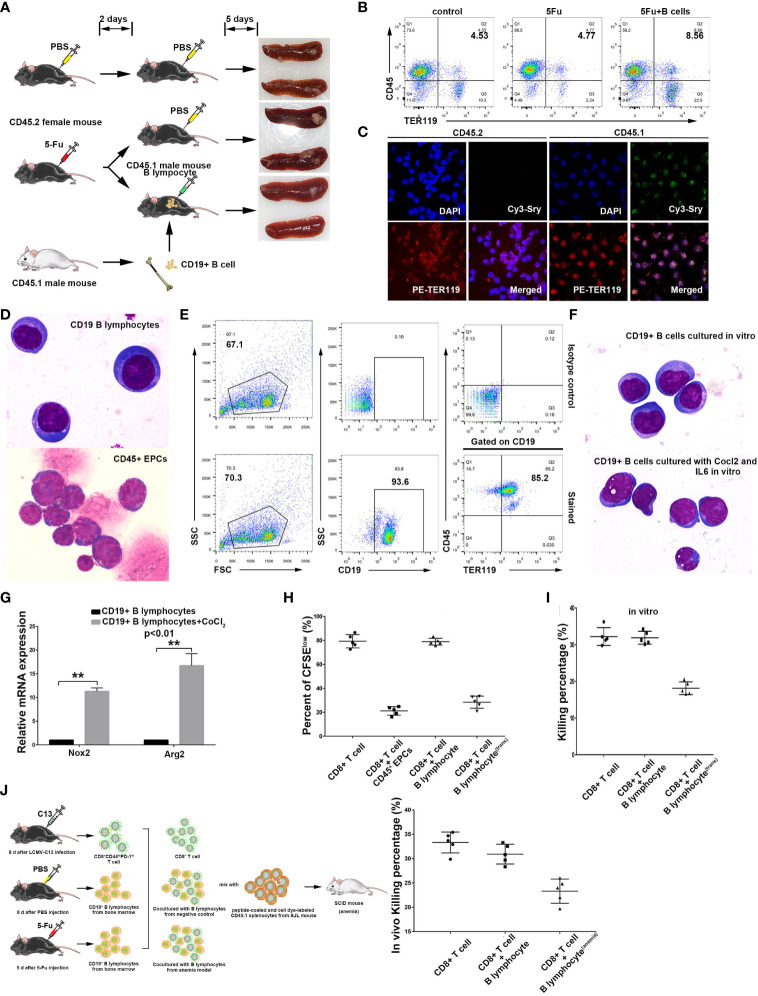
Induction of non-cancerous murine B lymphocytes into CD45^+^ erythroblast-like cells. **(A)** A schematic diagram showing B lymphocyte transdifferentiation into CD45^+^ EPCs in an anemic mouse model. Bone marrow B lymphocytes from CD45.1 male mice (SJL) were used for cell transplantation. CD45.2 female mice (C57BL6) were used for establishment of 5FU-mediated anemia and as receivers of B cells from CD45.1 male mice. **(B)** Detection flow cytometry of splenic CD45^+^ EPCs in anemic mice and anemic mice with B cell transplantation. Note: CD45^+^ EPCs were enriched in the spleens of anemic CD45.2 female mice. **(C)** Confocal microscopy showing purified CD45^+^ EPCs from the anemic CD45.2 female mouse spleen (with transplanted B cells). The cells from anemic mice were sorted by mouse CD45.1 and TER119 antibodies, and the CD45^+^ EPCs derived from B cells were detected via FISH with a probe against the male marker gene Sry. **(D)** Morphology of splenic B lymphocytes (CD19^+^ B lymphocytes from a healthy C57BL6 mouse) and CD45^+^ EPCs (CD45^+^TER119^+^) from an anemic C57BL6 mouse with magnification 1000×. **(E)** *In vitro* induction of TER119 expression in non-cancerous CD19^+^ B lymphocytes from mouse spleen by exposure to CoCl_2_ and IL6 at a concentration of 100 μM and 200 ng/ml, respectively. **(F)** Wright-Giemsa staining showing the morphology of CD19^+^ B lymphocytes and CD19^+^ B lymphocytes treated with CoCl_2_ (100 μM) and IL6 (200 ng/ml) *in vitro*. Magnification 1000×. **(G)** qRT-PCR showing the intracellular ROS-related genes Nox2 and Arg2 in normal B lymphocytes (control) and CoCl_2_/IL6-induced B lymphocytes (CD45^+^ TER119^+^) (n=3). **(H)** Inhibition of CD8^+^ T cell proliferation by CD45^+^ EPCs (CD45^+^TER119^+^CD71^+^ cells), B lymphocytes (CD19^+^ B cells), or B lymphocytes^trans^ (CoCl_2_/IL6-induced B lymphocytes). The proliferative capacity of CFSE-labeled CD8^+^ T cells in response to anti-CD3 and anti-CD28 was analyzed after co-culture with CD45^+^ EPCs, B lymphocytes, or B lymphocytes^trans^ at ratio of 1:2 (CD8^+^ T cells: tested cells) (n=5). **(I)** Inhibition of CD8^+^ T cell killing efficiency *ex vivo* by B lymphocytes or B lymphocytes^trans^ (CoCl_2_/IL6-induced B lymphocytes). B lymphocytes or B lymohocytes^trans^ were co-cultured with sorted CD8^+^ T cells (sorted from LCMV-C13 infected C57BL6 mice) and *ex vivo* T cell killing efficiency was determined after 6 hours (n=5). C57BL6 mouse splenocytes coated with GP33 were used as target cells. **(J)** Inhibition of CD8^+^ T cell killing efficiency *in vivo* by B lymphocytes or B lymphocytes^trans^ (CoCl_2_/IL6-induced B lymphocytes). B lymphocytes or B lymohocytes^trans^ were co-injected with sorted CD8^+^ T cells (sorted from LCMV-C13 infected C57BL6 mouse) and *in vivo* T cell killing efficiency was determined after 24 hours (n=5). C57BL6 mouse splenocytes coated with GP33 were used as target cells. The symbols ** means p<0.01.

It has been shown that immunosuppressive erythroid cells often appear with the occurrence of anemia (25). Anemia is a common condition among cancer patients, with some such cases being caused by chemotherapy (42, 43). To investigate the possibility of the transdifferentiation of non-cancerous B cells into CD45^+^ EPCs, we utilized 5-fluorouracil (5FU), a chemotherapeutic drug, to induce acute anemia in mice. Using 5Fu-mediated anemic mice (CD45.2 C57/BL6 female mice) as B cell transplantation recipients, we injected B lymphocytes from CD45.1 male mice into anemic CD45.2 female mice (**Figure 2A**) and observed significant enrichment of CD45^+^TER119^+^ cells in the spleen of anemic mice (**Figure 2B**; **Supplementary Figure 6A**). The VDJ rearrangement of these cells was similar to that of B cells (**Supplementary Figure 6B**). The cells were sorted based on CD45.1. *In situ* hybridization with a probe detecting Sry mRNA, a male gene product (44), together with TER119 staining revealed that the B lymphocytes transplanted from male mice underwent significant phenotypic modifications in anemic female mice (**Figure 2C**). Wright-Giemsa staining revealed the dissimilarity in morphology between CD45^+^TER119^+^ cells and B cells (**Figure 2D**).

Notably, B cells from anemic mice tend to transdifferentiate more readily when exposed to hypoxia, whereas B cells from normal mice fail to differentiate under similar hypoxic conditions (data not shown). These results suggest that there must be other inducing factors that cause the transdifferentiation of B cells *in vivo*. Serum constituent analysis revealed that the anemic cohort displayed a relatively high level of IL6 (**Supplementary Figure 6C**). This result indicates that the induction of CD45^+^ EPCs from B cells in anemic mice may also be related to infection. We therefore augmented IL6 in B cell culture and observed the transdifferentiation of B cells into CD45^+^TER119^+^ cells (**Figure 2E**), although no apparent morphological change occurred (**Figure 2F**). Moreover, the expression of NOX2 and Arg2, both of which are associated with ROS generation and activity, was upregulated in these cells (**Figure 2G**). The CD45^+^TER119^+^ cells derived from normal B cells possessed the ability to impede T cell proliferation and suppressed the killing efficiency of CD8^+^ T cells (**Figures 2H–J**; **Supplementary Figures 6D, E**). All these results confirm that immunosuppressive TER119-positive cells can be derived from normal B cells.

### Identification of immunosuppressive erythroblast-like cells from B cells in tumor-bearing and neonatal mice

**Figure 3:**
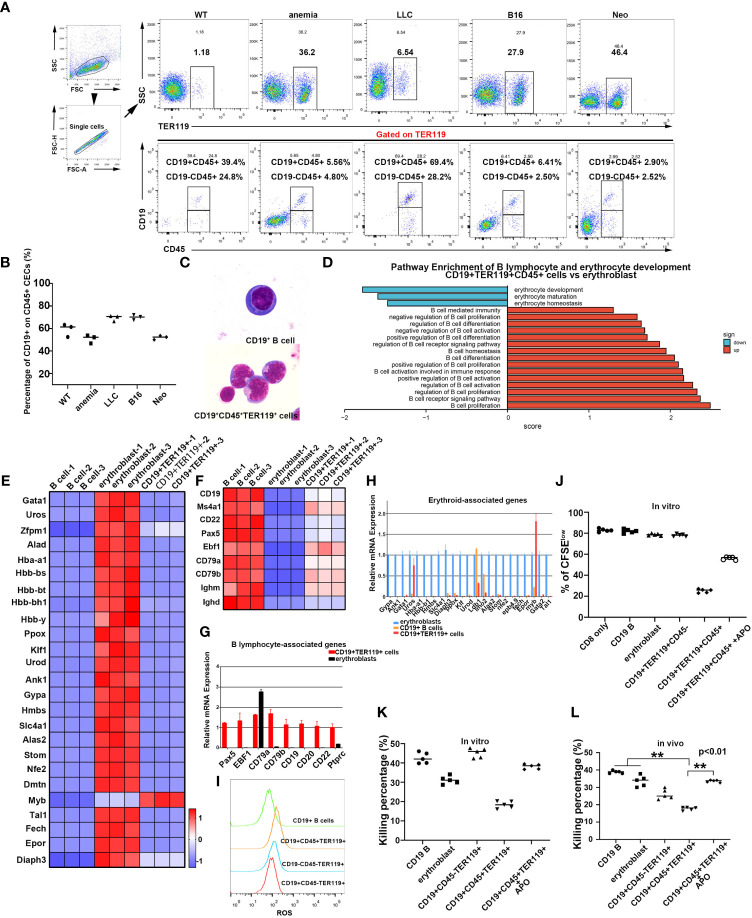
The discovery of CD19^+^TER119^+^ cells in mice. **(A)** Identification of CD19^+^TER119^+^CD45^+^ and CD19^-^TER119^+^CD45^+^ cells in mice. Flow cytometry analysis of CD19^+^TER119^+^CD45^+^ and CD19^-^TER119^+^CD45^+^ cells in spleens of C57BL6 mice under different physiological conditions: WT, normal C57BL6 mice; anemia, mice with PHZ-mediated acute hemolytic anemia; LLC and B16, tumor-bearing mice; Neo, neonatal mice (1-2 weeks). Representative flow cytometry and cumulative composite data show the frequency of CD19^+^CD45^+^ and CD19^-^CD45^+^ cells within the TER119+ population in the spleen of WT, anemic (4 days), tumor-bearing (LLC for 35 days and B16 for 21 days), and neonatal (within two weeks after birth) mice. **(B)** Cumulative composite data showing the proportion of CD19^+^TER119^+^CD45^+^ cells in CD45^+^ EPCs (CD45^+^TER119^+^) within the spleens under different physiological conditions. Mature red blood cells were removed. **(C)** Wright-Giemsa showing the morphology of mouse CD19^+^ B cells and natural CD19^+^TER119^+^CD45^+^ cells (as distinct from CoCl_2_/IL6-induced CD19^+^ B cells) with magnification 1000×. **(D)** Pathway enrichment analysis performed via GSEA. Significantly enriched items (nominal *p <*0.05) in CD19^+^TER119^+^CD45^+^ cells from the spleens of neonatal mice compared with the erythroblasts from the livers of 14.5-day mouse embryos are shown with enrichment scores. **(E)** Heat map illustrating the relative expression of erythroid cell-associated genes in B cells, erythroblasts, and CD19^+^TER119^+^ cells. **(F)** Heat map illustrating the relative expression of B cell-associated genes in B cells, erythroblasts, and CD19^+^TER119^+^ cells. **(G)** qPCR showing the expression of B lymphocyte-associated marker genes in B cells, erythroblasts, and CD19^+^TER119^+^ cells (n=3). **(H)** qPCR showing the expression of erythroid cell-associated genes in B cells, erythroblasts, and CD19^+^TER119^+^ cells (n=3). **(I)** Intracellular ROS production in CD19^+^ B cells, CD19^+^TER119^+^CD45^+^ cells, CD19^-^TER119^+^CD45^-^ cells, and CD19^+^CD45^-^TER119^+^ cells as analyzed *via* flow cytometry. **(J)** Inhibition of CD8^+^ T cell proliferation by CD19^+^ B cells, erythroblasts, CD19^+^TER119^+^CD45^-^ cells, CD19^+^TER119^+^CD45^+^ cells, or CD19^+^TER119^+^CD45^+^ (APO-induced ROSi) cells. The proliferative capacity of CFSE-labeled CD8^+^ T cells in response to anti-CD3 and anti-CD28 was analyzed after co-culture with CD19^+^ B cells, erythroblasts, CD19^+^TER119^+^CD45^-^ cells, CD19^+^TER119^+^CD45^+^ cells, or CD19^+^TER119^+^CD45^+^ (APO-induced ROSi) cells at ratio of 1:2 (CD8^+^ T cells: tested cells). **(K)** Inhibition of CD8^+^ T cell killing efficiency *in vitro* by CD19^+^ B cells, erythroblasts, CD19^+^TER119^+^CD45^-^ cells, CD19^+^TER119^+^CD45^+^ cells, or CD19^+^TER119^+^CD45^+^ (APO-induced ROSi) cells. The tested cells were co-cultured with sorted CD8^+^ T cells (at ratio of 2:1) and *in vitro* T cell killing efficiency was determined after 6 hours (n=5). **(L)** Inhibition of CD8^+^ T cell killing efficiency *in vivo* by CD19^+^ B cells, erythroblasts, CD19^+^TER119^+^CD45^-^ cells, CD19^+^TER119^+^CD45^+^ cells, or CD19^+^TER119^+^CD45^+^ (APO-induced ROSi) cells. CD19^+^ B cells, erythroblasts, CD19^+^TER119^+^CD45^-^ cells, CD19^+^TER119^+^CD45^+^ cells, or CD19^+^TER119^+^CD45^+^ (APO-induced ROSi) cells were co-injected with sorted CD8^+^ T cells and *in vivo* T cell killing efficiency was determined after 24 hours (n=5). The symbols ** means p<0.01.

CD45^+^ EPCs exist in tumor-bearing and neonatal mice (22, 25). Consistent with these findings, we detected CD45^+^ EPCs in the spleens of mice transplanted with lung carcinoma and melanoma cells (**Figure 3A**), and a subset of CD45^+^ cells expressed both CD19 and TER119 markers (**Figure 3B**). The CD45^+^CD19^+^TER119^+^ cells from the spleens of tumor-bearing mice and B lymphocytes from normal mice differed in morphology (**Figure 3C**; the ratio of CD19^+^ CD45^+^, CD19^-^CD45^+^, and CD19^-^CD45^-^ cells in each group is shown in **Supplementary Figures 7A, B**). The immunosuppressive function of these CD45^+^CD19^+^TER119^+^ cells was observed, akin to the findings of Zhu et al. regarding CD45^+^ EPCs (**Supplementary Figures 7C–F**) (25). CD45^+^ EPCs are widely believed to be a byproduct of later-stage tumors due to anemia. However, CD45^+^CD19^+^TER119^+^ cell populations were also detected in the spleens of normal neonatal mice (**Figure 3A**).

We subsequently sorted these cells and conducted transcriptome sequencing (**Supplementary Figure 8**; GEO accession code: GSE201937). Bioinformatics analysis ascertained that CD45^+^CD19^+^TER119^+^ cells exhibited similar pathway enrichment to the TER cells described by Han et al. and by Zhao et al. (25, 26). Moreover, the CD45^+^CD19^+^TER119^+^ cells exhibited distinctive enhancement in pathways linked to B cells and a negative correlation with erythroid pathways when compared to the erythroblasts from 14.5-day fetal livers (**Figure 3D**). We further performed analyses of our transcriptome data derived from B cells (sorted from adult spleens of normal mice), erythroblasts (sorted from 14.5-day fetal livers), and CD45^+^CD19^+^TER119^+^ cells (sorted from neonatal mouse spleens within two weeks after birth). The CD45^+^CD19^+^TER119^+^ cells exhibited a stronger proclivity toward B cell differentiation rather than erythroid cell formation (**Figures 3E, F**). The accuracy of the sequencing results was partially validated by quantitative PCR (**Figures 3G, H**). To ascertain the functional similarity between CD45^+^CD19^+^TER119^+^ cells and CD45^+^ EPCs, an assessment of intracellular ROS content was conducted. CD45^+^CD19^+^TER119^+^ cells exhibited significantly higher levels of intracellular ROS compared to CD19^+^ B cells, CD45^-^CD19^-^TER119^+^ cells (erythroblasts), and CD45^-^CD19^+^TER119^+^ cells (**Figure 3I**). Compared with B cells, CD45^+^CD19^+^TER119^+^ cells were enriched with the ROS generation pathway (**Supplementary Figure 9**).

Interestingly, CD45^-^CD19^+^TER119^+^ TER cells (26) were also found in neonatal mouse spleens; these were morphologically similar to B cells and CD45^+^CD19^+^TER119^+^ cells (**Supplementary Figures 10A, B**). These CD45^-^CD19^+^TER119^+^ cells expressed artemin protein (**Supplementary Figures 10C, D**), which is consistent with the TER cells described by Han et al. (26). These findings suggest that even the TER cells described by Han et al. might be B cells or derived from B cells. *In vitro* functional assays verified the capacity of CD45^+^CD19^+^TER119^+^ cells to suppress the proliferation and immune function of CD8^+^ T cells (**Figures 3J, K**; **Supplementary Figure 11A, B**). Moreover, these suppressive activities could be alleviated by eliminating intracellular ROS (**Figures 3J, K**; **Supplementary Figures 11A, B**). The CD45^+^CD19^+^TER119^+^ cells were also identified as hindering the destruction of target cells by CD8^+^ T cells through the generation of ROS (**Figure 3L**; **Supplementary Figure 11C**), which is consistent with the results obtained from *in vitro* experiments. These data indicate that CD45^+^CD19^+^TER119^+^ cells exhibit an immunosuppressive function, in the same way as the CD45^+^ EPCs. Taken together, our results confirm that B cells have the potential to transdifferentiate into erythroblast-like immune-inhibiting cells under specific physiological and pathological conditions.

### Identification of CD235a^+^CD19^+^ double-positive cells in peripheral blood from patients with leukemia

**Figure 4:**
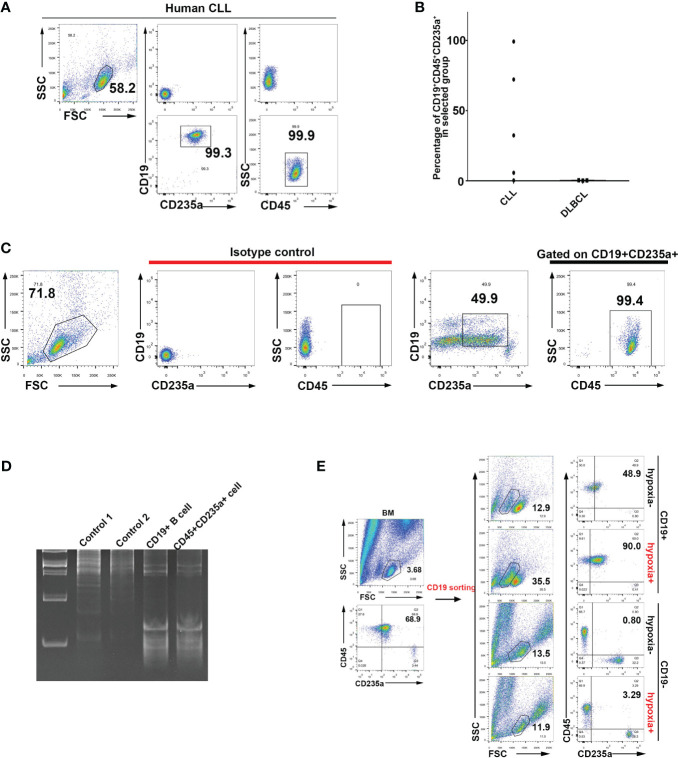
Identification of human B lymphocytes undergoing transdifferentiation into CD45^+^ EPCs. **(A)** Identification via flow cytometry of CD19^+^CD235a^+^CD45^+^ cells in peripheral blood mononuclear cells (PBMCs) from CLL patients. **(B)** Statistical analysis of CD19^+^CD235a^+^CD45^+^ cells in PBMCs of patients diagnosed with CLL and DLBCL. **(C)** Identification of CD19^+^CD235a^+^CD45^+^ cells in PBMCs from an AML patient. **(D)** Analysis of VDJ rearrangement in CD19^+^ B cells and CD45^+^CD235a^+^ cells from bone marrow of an MDS patient. Control 1: genomic DNA from 293T cells (negative control); control 2: genomic DNA from HCT116 cells (negative control); CD19^+^ B cell: genomic DNA from CD19^+^ B cells in bone marrow from an MDS patient; CD45^+^CD235a^+^ cell: genomic DNA from CD45^+^CD235a^+^ cells in bone marrow from the same MDS patient. **(E)** Flow cytometry analysis of hypoxia-induced transdifferentiation of CD19^+^ B cells isolated from bone marrow of MDS patients. CD19^+^ B cells were sorted from bone marrow of MDS patients and cultured with hypoxia-induced differentiation *in vitro* (CoCl_2_-induced hypoxia). CD19-postive (CD19^+^) cells are more likely to undergo CD45^+^CD235a^+^ transdifferentiation under induction of hypoxia. CD19-negative (CD19^-^) cells were used as controls and subjected to the same hypoxia induction treatment.

TER119 is considered to be a specific cell surface marker of mouse erythroid cells, while CD235a is a TER119-like molecule that is considered to be specifically expressed on the surface of human erythroid cells. To investigate whether human TER cells express CD19, we harvested blood samples from patients with various hematopoietic malignancies and analyzed the mononuclear cells (PBMCs) after lysis of mature RBCs. Strikingly, three out of five chronic lymphocytic leukemia (CLL) blood samples contained a large proportion of CD45^+^/CD19^+^/CD235a^+^ cells (**Figures 4A, B**; **Supplementary Figure 12A**). As a negative control, diffuse large B cell lymphoma (DLBCL) blood was shown to contain almost no CD45^+^/CD19^+^/CD235a^+^ cells (**Figure 4B**; **Supplementary Figure 12B**), possibly because no tumor cells were available in peripheral blood at the time of sample collection. Two out of five cases of CLL contained only a small number of CD45^+^/CD19^+^/CD235a^+^ cells (**Figure 4B**; **Supplementary Figure 12A**), most likely due to the elimination of tumor cells by chemotherapy. In one case of peripheral blood from an AML patient, CD45^+^/CD19^+^/CD235a^+^ TER cells clearly existed, although only a small proportion of such TER cells were strongly CD19-positive, and the remaining cells were weakly CD19-positive (**Figure 4C**). Another three cases of AML, one case of AML-ml, one case of AML-M4b-CR1, one case of MDS-EB-1, one case of MDS-SLD, one case of MM, one case of AL, and one case of B-ALL produced small numbers of or no CD19^+^/CD235a^+^ cells at the time of sample collection (data not shown). The CD45^+^ EPCs that were isolated from the bone marrow of the same patient exhibited VDJ rearrangement fragments that were akin to those observed in B cells (**Figure 4D**). Upon conducting CD19 sorting of bone marrow cells sampled from a patient with myelodysplastic syndrome (MDS), a comparative analysis confirmed the presence of CD45^+^CD235a^+^ cells (**Figure 4E**). This finding indicated that CD19^+^ cells exhibit the potential to undergo transdifferentiation into CD45^+^CD235a^+^ cells when subjected to hypoxic stimulation. In addition, CLL patients all exhibited strong infection symptoms, including the two patients with the highest proportion of CD45^+^/CD19^+^/CD235a^+^ cells, who died six months after undergoing CD45/CD19/CD235a testing, and one patient with approximately 30% CD45^+^/CD19^+^/CD235a^+^ cells, whose tumor recurred with pulmonary infection one year after CD45/CD19/CD235a testing. We found that some CD63^+^ erythroid precursors also exhibit CD19 expression, and CD63^+^ erythroblasts are considered to have an immune regulatory function (**Supplementary Figure 13**) (45). All these results indicate that, like murine B-lymphoid cells, human B-lymphoid cells also exhibit spontaneous acquisition of erythroid lineage markers. These results also suggest that the presence of CD45^+^/CD19^+^/CD235a^+^ cells in tumor patients might be associated with poor prognosis and increased risk of infection.

## Discussion

Recent studies have discovered a subset of erythroblast-like cells that modulate the immune system and the tumor microenvironment (23, 24, 27, 28). Erythroid cells are usually considered to be functionally homogenous, whereas B cells have been confirmed to be a class of cell populations that are markedly heterogeneous. In the current study, we found that a proportion of CD45^+^ EPCs (25, 28) and CD45^-^ TER cells (26), both of which were considered to be erythroid cells, were either B lymphocytes or originated from B cells; this was the case both in mice and in human patients. A mechanistic understanding of the transdifferentiation of B cells to TER cells could be of future benefit in therapeutic settings. Determination of which types of anemia, either inherited or acquired, stimulate and sustain the expansion of TER cells is also important.

The presence of CD45^+^/CD19^+^/TER119^+^ cells among CD45^+^ EPCs may result in their misidentification as conventional erythroid cells, thereby compromising the accuracy of differentiation analyses and subsequent analyses. This is especially true given that, based on published sequencing data, many B-cell development-related genes are expressed in CD45^+^ EPCs and CD45^-^ TER cells (**Supplementary Figures 3–5**) (25, 26). The presence of representative markers such as CD74 (46), CD63, and CD45, which are commonly found in B cells, also indicates the likelihood of a B cell origin for these immunosuppressive erythroblast-like cells. Therefore, the CD19 marker should be routinely used in laboratory testing to distinguish whether such cells are B lymphocytes, classical erythroblasts, or immunosuppressive TER cells (whether they are CD45^+^ or not), especially under conditions of hypoxia or anemia.

In summary, some immunosuppressive erythroblasts are transdifferentiated from a subset of CD19^+^ B lymphocytes; this result emphasizes the plasticity of B cells. Our findings imply that targeting a specific set of B cells (CD19^+^/TER119^+^ cells in mice and CD19^+^/CD235a^+^ cells in humans) instead of erythroblasts should restore adaptive immunity. Our findings also suggest that CD19 should be included when purifying conventional erythroid cells, since TER119- or CD235a-positive “red” cells might not be genuine red cells.

## Data availability statement

The datasets presented in this study can be found in online repositories. The names of the repository/repositories and accession number(s) can be found below: https://www.ncbi.nlm.nih.gov/geo/, GSE201915; https://www.ncbi.nlm.nih.gov/geo/, GSE201937.

## Ethics statement

The studies involving human participants were reviewed and approved by Ethics Committee of Medical College of Yangzhou University Ethics Committee of Yangzhou Subei People’s Hospital. The patients/participants provided their written informed consent to participate in this study. The animal study was reviewed and approved by Ethics Committee of Medical College of Yangzhou University. Written informed consent was obtained from the individual(s) for the publication of any potentially identifiable images or data included in this article.

## Author contributions

DY and ZY designed the study. ZY, ZW, LW, and YW performed the experiments and analyzed the data. ZX performed the bioinformatics analyses. FW, ZY, ZW, and YL assisted in clinical sample collection and handling. ZY drafted the manuscript and DY revised the manuscript. All authors contributed to the article and approved the submitted version.
