# Supplementary material for: B lymphocytes transdifferentiate into immunosuppressive erythroblast-like cells
Source: Front Immunol. 2023 Jul 21;14:1202943. doi: 10.3389/fimmu.2023.1202943 (PMC10401433; doi:10.3389/fimmu.2023.1202943)
Supplement: Supplementary file 1 [file DataSheet_1.docx]

**Supplemental Figures and Figure legends**

**Supplemental Figure 1. Construction of BL1 cell line and its differentiation into BL1-TER119 cells**

1. The schematic diagram of the establishment of murine BL1 B lymphoma cell line.
2. Immunofluorescence of BL1-TER119 cells.
3. Genomic PCR showing the gag-myc (identifying the myc sequence engineered in the expression retrovirus) and VJ558-JH4 (identifying the VDJ rearrangement in B lymphocytes) fragments in both BL1-CD19 and BL1-TER119 cells.
4. Enrichment plot of the immune regulation-related pathways between BL1-TER119 cells and BL1-CD19 cells.
5. The mRNA levels of ROS pathway gene Nox2, Arg2 and Nrf2 in BL1-TER119 and BL1-CD19 cells analyzed by quantitative real-time PCR (qRT-PCR).
6. The influence of hydrogen peroxide stimulation on hemoglobin production in BL1-TER119 cells. The hemoglobin expression in H_2_O_2_-induced groups (H_2_O_2_ treatment concentration were 5 μM, 10 μM, 15 μM, 20 μM, 25μM and 0 μM used as negative control) was identified by Western Blot.
7. The production of hemoglobin and HIF1α protein was investigated using Western Blot. Note: the levels of hemoglobin and HIF1α were observed to be directly proportional to the concentration of CoCl_2_.
8. Immunohistochemistry of TER119 in CoCl_2-_induced BL1-CD19 cells.
9. The effect of HIF pathway inhibitors (Lw6) on ROS production in BL1-TER119 cells analyzed by flow cytometry.
10. The effect of HIF pathway inhibition on ROS production in BL1-TER119 cells analyzed by flow cytometry.
11. Statistical analysis of the effect of HIF pathway inhibition on ROS production in BL1-TER119 cells.

**Supplemental Figure 2. Functional analysis of BL1 cell line and the transdifferentiation of other lymphoma cell lines.**

1. The effect of HIF pathway inhibitors (Lw6) on TER119 expression in BL1-TER119 cells analyzed by flow cytometry.
2. The effect of HIF pathway inhibition on TER119 expression in BL1-TER119 cells analyzed by flow cytometry (histogram).
3. Statistical analysis of the effect of HIF pathway inhibition on TER119 expression in BL1-TER119 cells.
4. CFSE staining and flow cytometry showing the proliferation rate of CD8^+^ T cells after coculture with BL1-CD19, BL1-TER119, BL1-CD19+ROSi, and BL1-TER119+ROSi.
5. qRT-PCR measuring the mRNA levels of erythroblast-related genes and B lineage-specific gene Pax5 and EBF1 in BL1-CD19 and BL1-TER119 cells.
6. Flow cytometric analysis of TER119 and CD71 expression in different murine cells. 38B9 and A20 are two mouse B lymphoma cell lines and Myc5 is a B lymphoma cell-derived myeloid cell line. Note: Myc5 is TER119 positive.

**Supplemental Figure 3. The B cell marker gene expression and Enrichment plot of B cell associated pathways in GEO database (Accession code: GSE106384).**

**Supplemental Figure 4. Pathway enrichment analysis performed with GSEA. The enrichment pathways of B lymphocyte and erythrocyte development in GEO database were analyzed (Accession code: GSE106384).**

**Supplemental Figure 5. Pathway enrichment analysis performed with GSEA. The enrichment pathways of B lymphocyte and erythrocyte development in GEO database were analyzed (Accession code: GSE109429). B cell and erythroid cell associated genes in GSE109429 were displayed.**

**Supplemental Figure 6. Induction and identification of CD45^+^ EPCs from mouse non-neoplastic B cells.**

1. Statistically analysis of the proportion of CD45^+^TER119^+^ cells in the spleens of 5Fu-induced anemia models.
2. PCR analysis of VDJ rearrangement in mouse CD45^+^ EPCs.
3. Serum IL6 levels in 5Fu-induced anemic mice.
4. CFSE staining and flow cytometry showing the proliferation rate of CD8^+^ T cells after coculture with CD45^+^ EPCs, CD19^+^ B cells, and CD19^+^ B-trans cells.
5. Representative flow cytometric analysis showing the immunosuppressive effect of CD19^+^ B^-trans^ cells, and CD45^+^ EPCs on the cell killing ability of CD8^+^ T cells both in vitro and in vivo. Note, the CFSE^low^ cells were splenocytes with no GP33 peptide-coated and the CFSE^high^ cells were those coated with GP33 peptide. CD19^+^ B cells were used as negative control.

**Supplemental Figure 7. Proportion and cellular immunosuppressive function of CD19^+^TER119^+^CD45^+^ cells in mouse spleens.**

1. Statistics of the proportion of CD19^+^CD45^+^TER119^+^ cells, CD19^-^CD45^+^TER119^+^ cells and CD19^-^CD45^-^TER119^+^ cells in spleens (n=3).
2. Statistics of the proportion of CD19^+^/CD19^-^CD45^+^TER119^+^ cells (CD45^+^ EPCs) and CD19^-^CD45^-^TER119^+^ cells in spleens (n=3).
3. CFSE staining and flow cytometry showing the proliferation rate of CD8^+^ T cells after coculture with CD19^+^TER119^+^CD45^+^ cells.
4. Statistical analysis of CD8^+^ T cell proliferation inhibition with CD19^+^TER119^+^CD45^+^ cells.
5. Representative flow cytometric analysis showing the immunosuppressive effect of CD19^+^TER119^+^CD45^+^ cells on the cell killing ability of CD8^+^ T cells in vivo.
6. Statistical analysis of the immunosuppressive effect of CD19^+^TER119^+^CD45^+^ cells on the cell killing ability of CD8^+^ T cells.

**Supplemental Figure 8. Sorting strategy of the CD19^+^TER119^+^CD45^+^ cells from neonatal mouse spleens.**

**Supplemental Figure 9. KEGG showing the genes in chemical carcinogenesis-reactive oxygen species (mmu05208) pathway differentially expressed in CD19^+^ B cells and CD19^+^TER119^+^CD45^+^ cells.**

**Supplemental Figure 10. The CD19^+^TER119^+^CD45^-^ cells identified in neonatal mouse spleens.**

1. The CD19^+^TER119^+^CD45^-^ cells were identified by flow cytometry.
2. Wright-Giemsa staining showing the morphology of CD19^+^ B cells, CD19^+^TER119^+^CD45^+^ cells, and CD19^+^TER119^+^CD45^-^ cells.
3. qRT-PCR showing the expression of artemin in CD19^+^TER119^+^CD45^-^ cells, CD19^-^TER119^+^CD45^-^ erythrocytes, CD19^+^TER119^+^CD45^+^ cells and CD19^+^ B cells.
4. Western Blot analysis of the artemin protein expression in CD19^-^TER119^+^CD45^-^ erythrocytes, CD19^+^ B cells, CD19^+^TER119^+^CD45^+^ cells, and CD19^+^TER119^+^CD45^-^ cells.

**Supplemental Figure 11. The immunosuppressive effect of CD19^+^CD45^+^TER119^+^ cells on the CD8^+^ T cells both in vitro and in vivo.**

1. CFSE staining and flow cytometry showing the proliferation rate of CD8^+^ T cells after coculture with CD19^+^ B cells, CD19^-^TER119^+^CD45^-^ erythroid cells, CD19^+^TER119^+^CD45^-^ cells, CD19^+^TER119^+^CD45^+^ cells, and CD19^+^TER119^+^CD45^+^ cells with ROSi.
2. Representative flow cytometric analysis showing the immunosuppressive effect of CD19^+^ B cells, CD19^-^TER119^+^CD45^-^ erythroid cells, CD19^+^TER119^+^CD45^-^ cells, CD19^+^TER119^+^CD45^+^ cells, and CD19^+^TER119^+^CD45^+^ cells with ROSi on the cell killing ability of CD8^+^ T cells in vitro.
3. Representative flow cytometric analysis showing the immunosuppressive effect of CD19^+^ B cells, CD19^-^TER119^+^CD45^-^ erythroid cells, CD19^+^TER119^+^CD45^-^ cells, CD19^+^TER119^+^CD45^+^ cells, and CD19^+^TER119^+^CD45^+^ cells with ROSi on the cell killing ability of CD8^+^ T cells in vivo.

**Supplemental Figure 12. The peripheral blood from CLL and DLBCL patients were analyzed with flow cytometry for the expression of CD19, CD235a and CD45.**

**Supplemental Figure 13. Detection of CD63^+^CD19^+^CD235a^+^ cells in peripheral blood from CLL patients.**
