# Supplementary material for: B lymphocytes transdifferentiate into immunosuppressive erythroblast-like cells
Source: Front Immunol. 2023 Jul 21;14:1202943. doi: 10.3389/fimmu.2023.1202943 (PMC10401433; doi:10.3389/fimmu.2023.1202943)
Supplement: Supplementary file 2 [file Image_1.pdf]

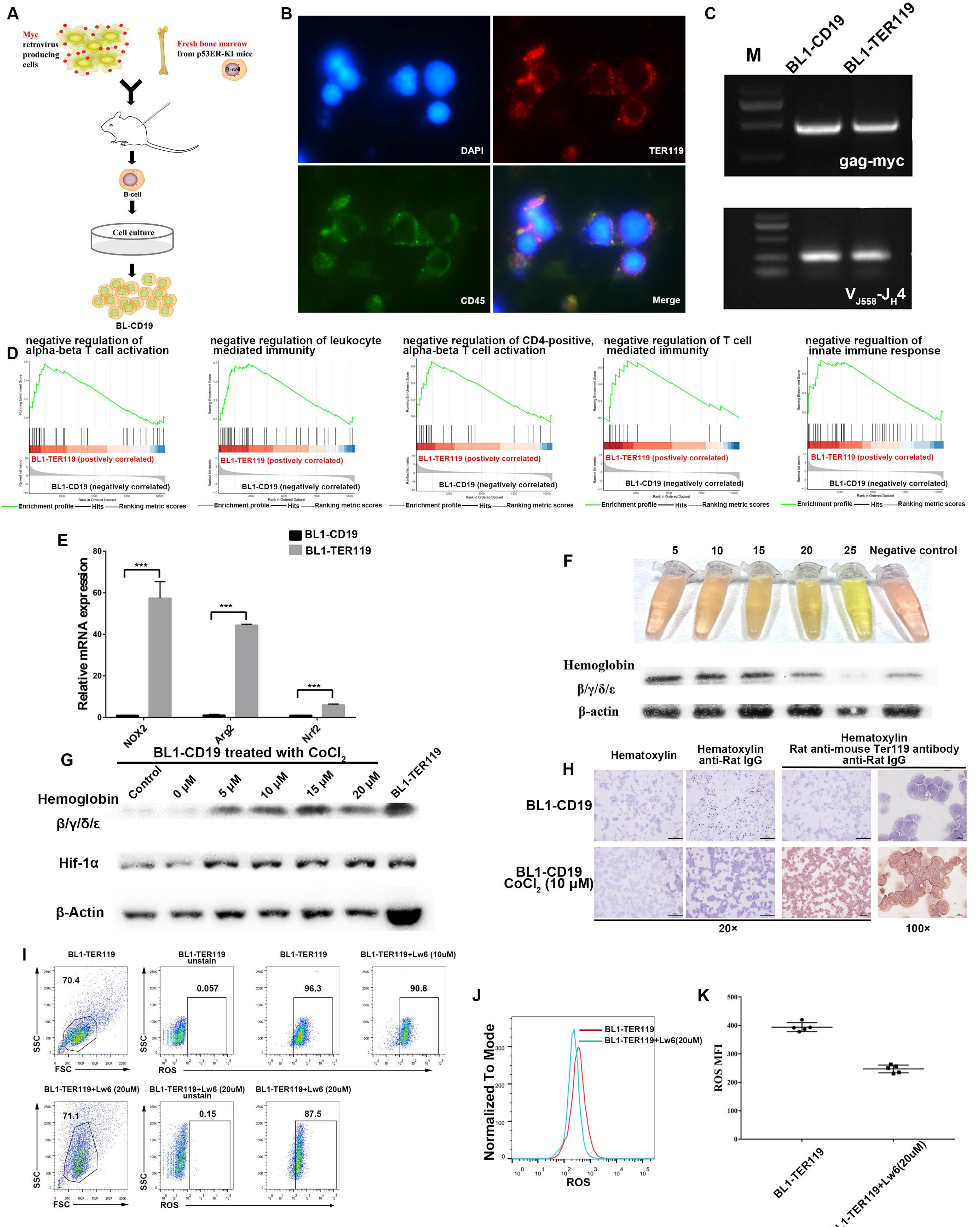

Supplemental Figure 1

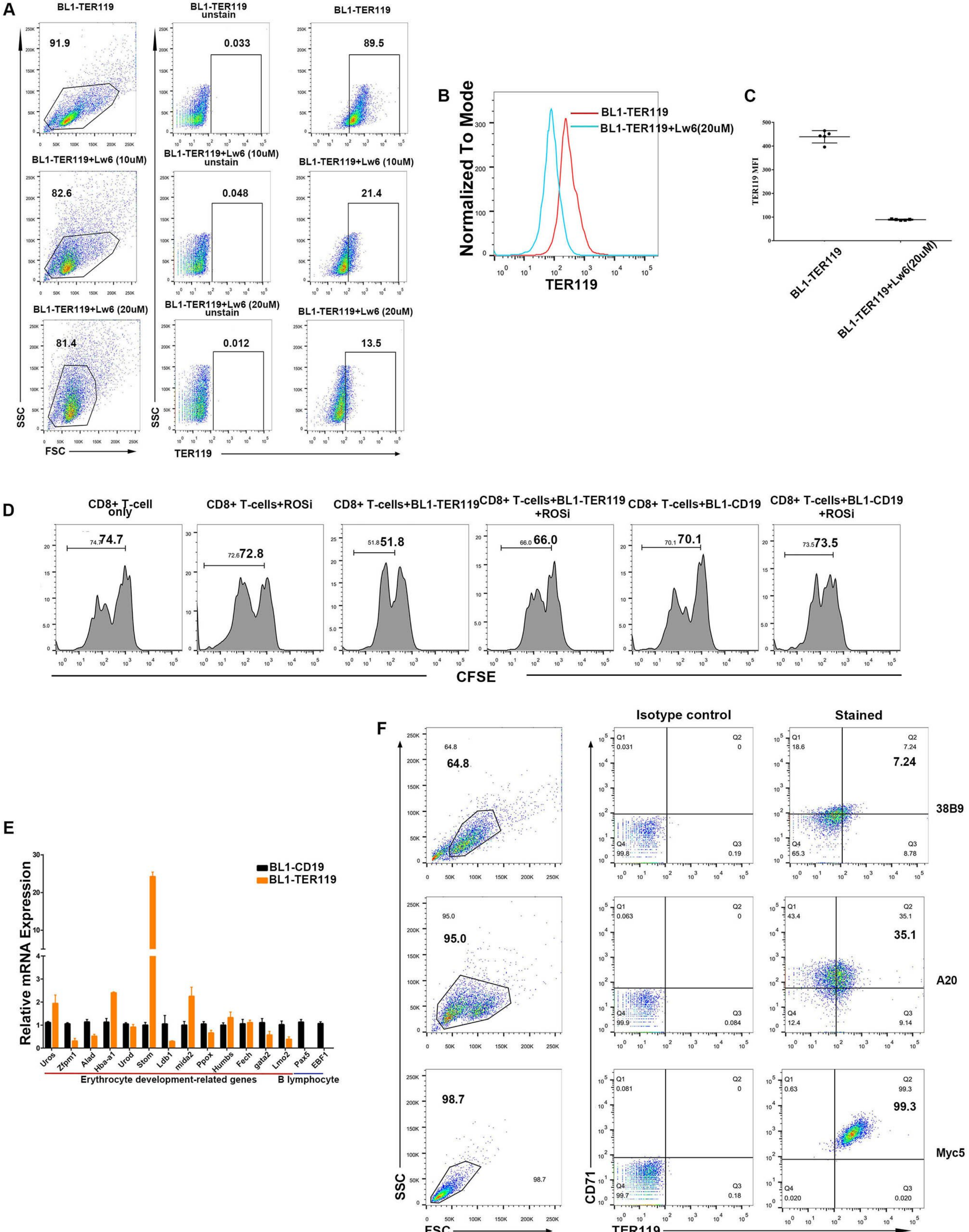

Supplemental Figure 2

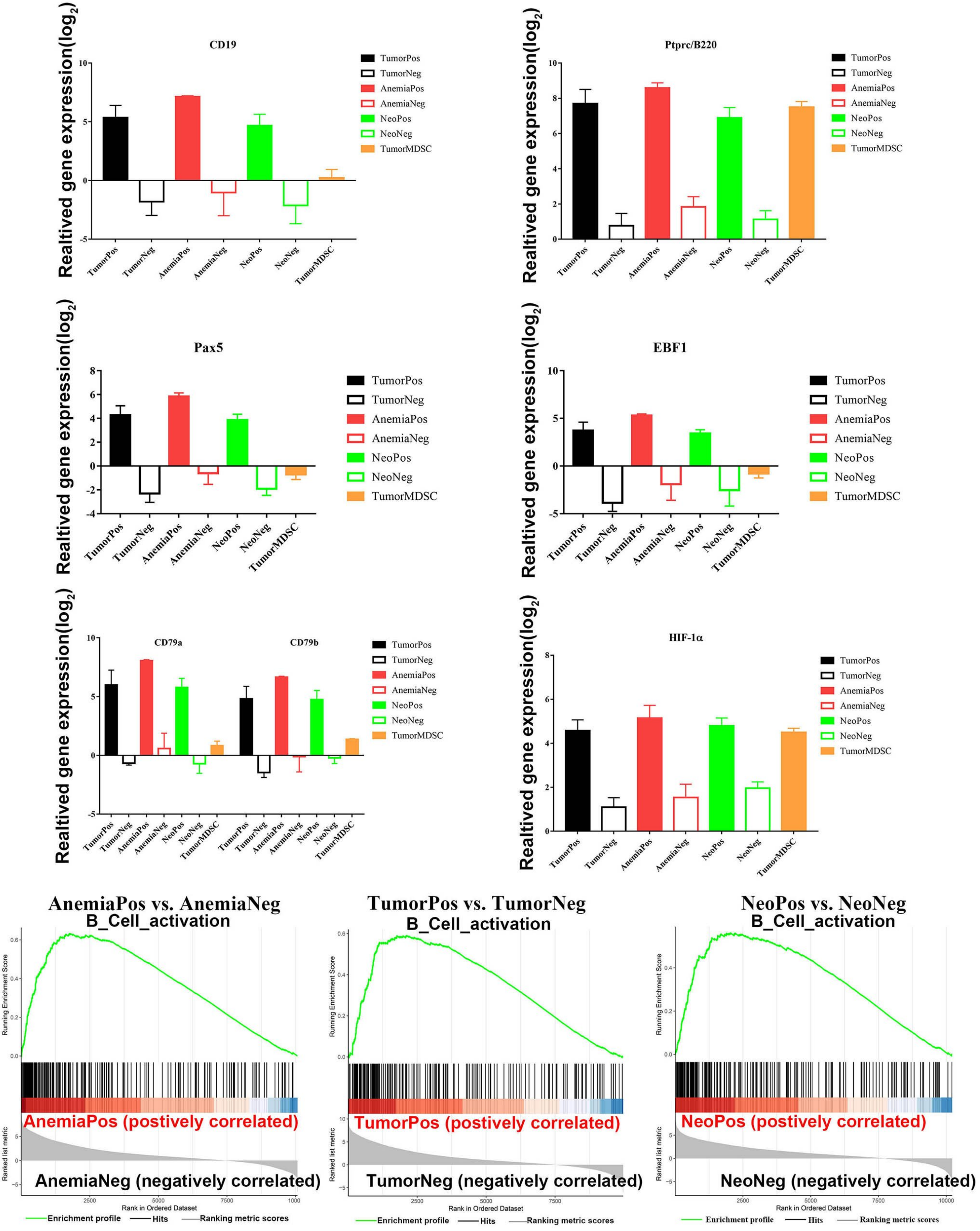

Supplemental Figure 3

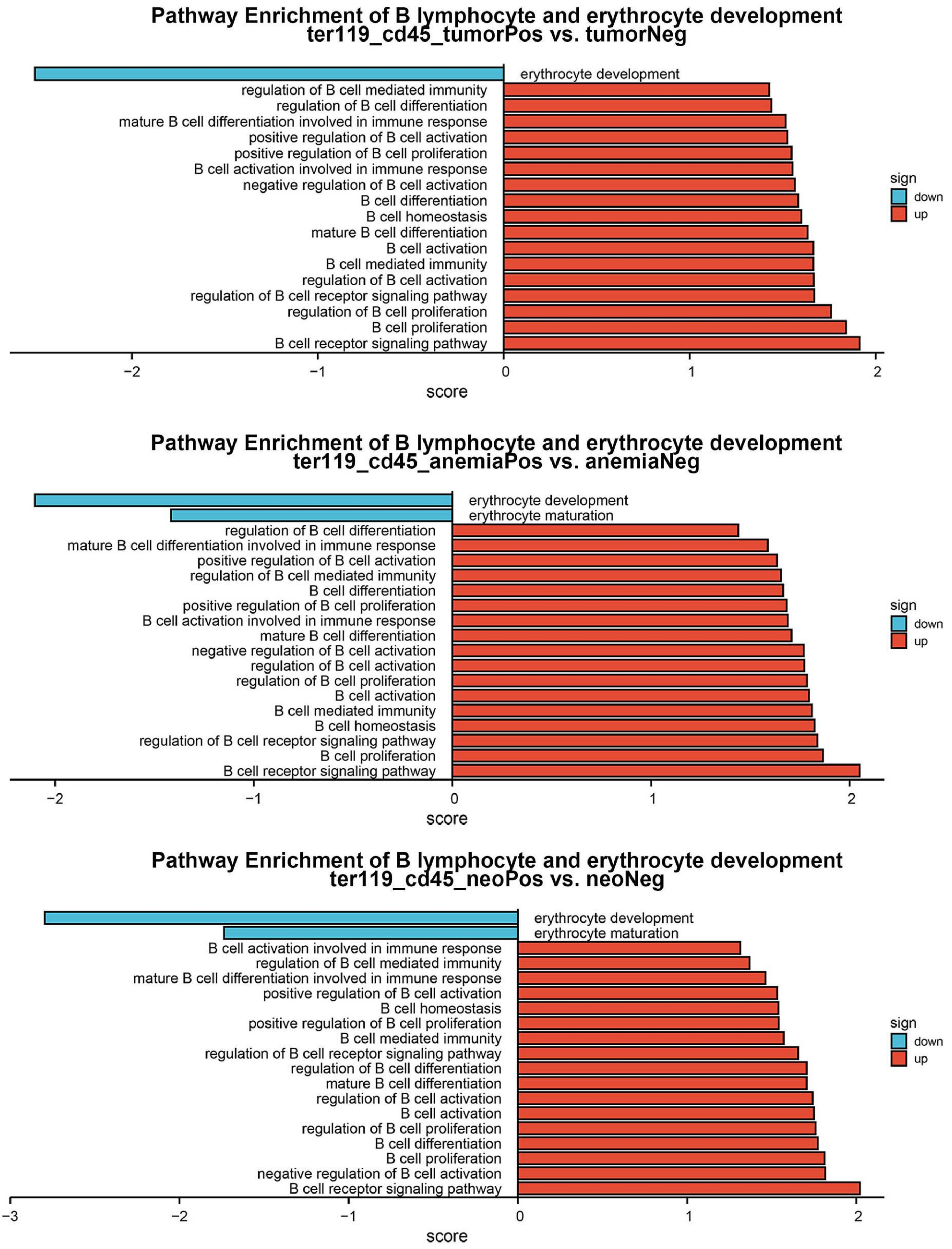

Supplemental Figure 4

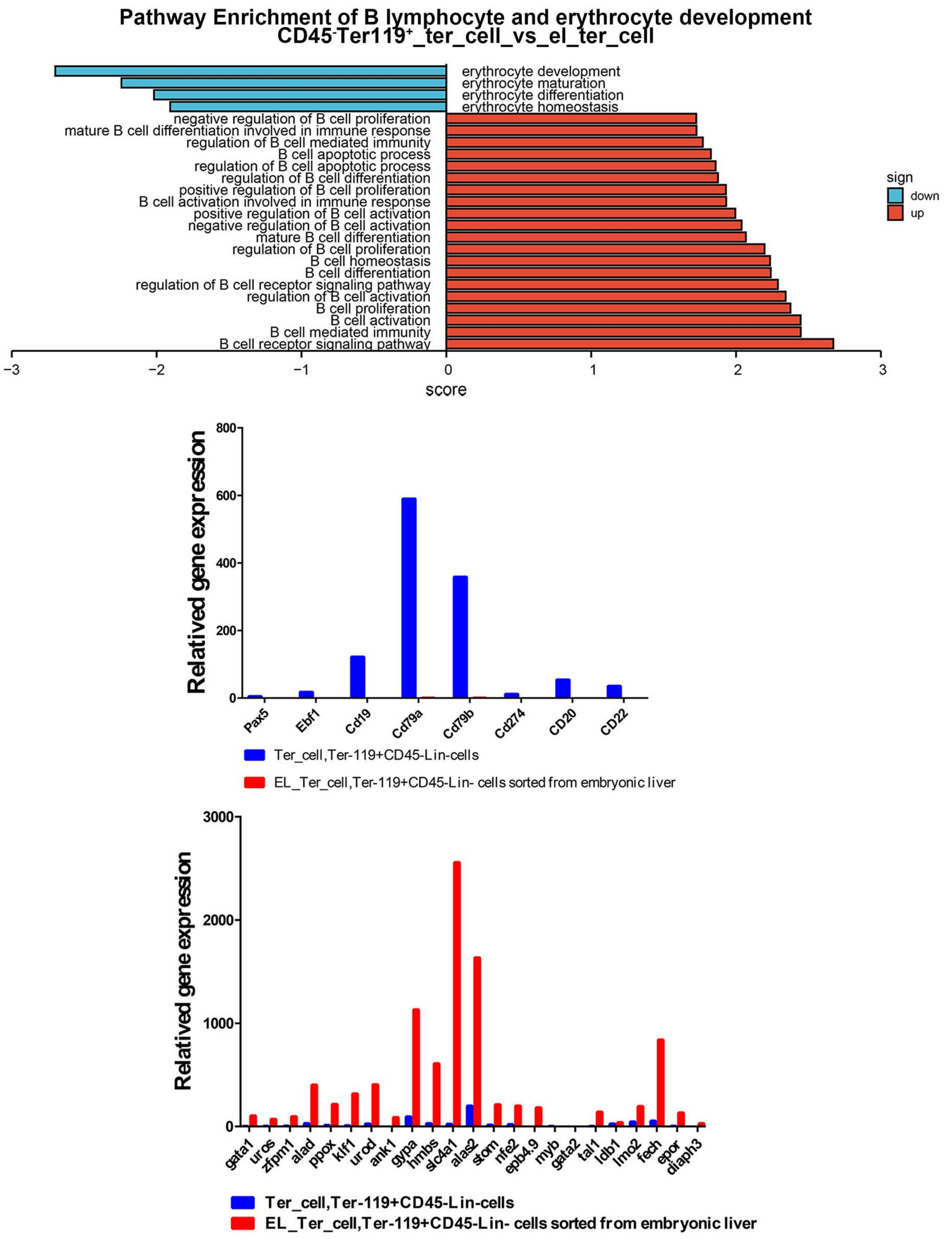

Supplemental Figure 5

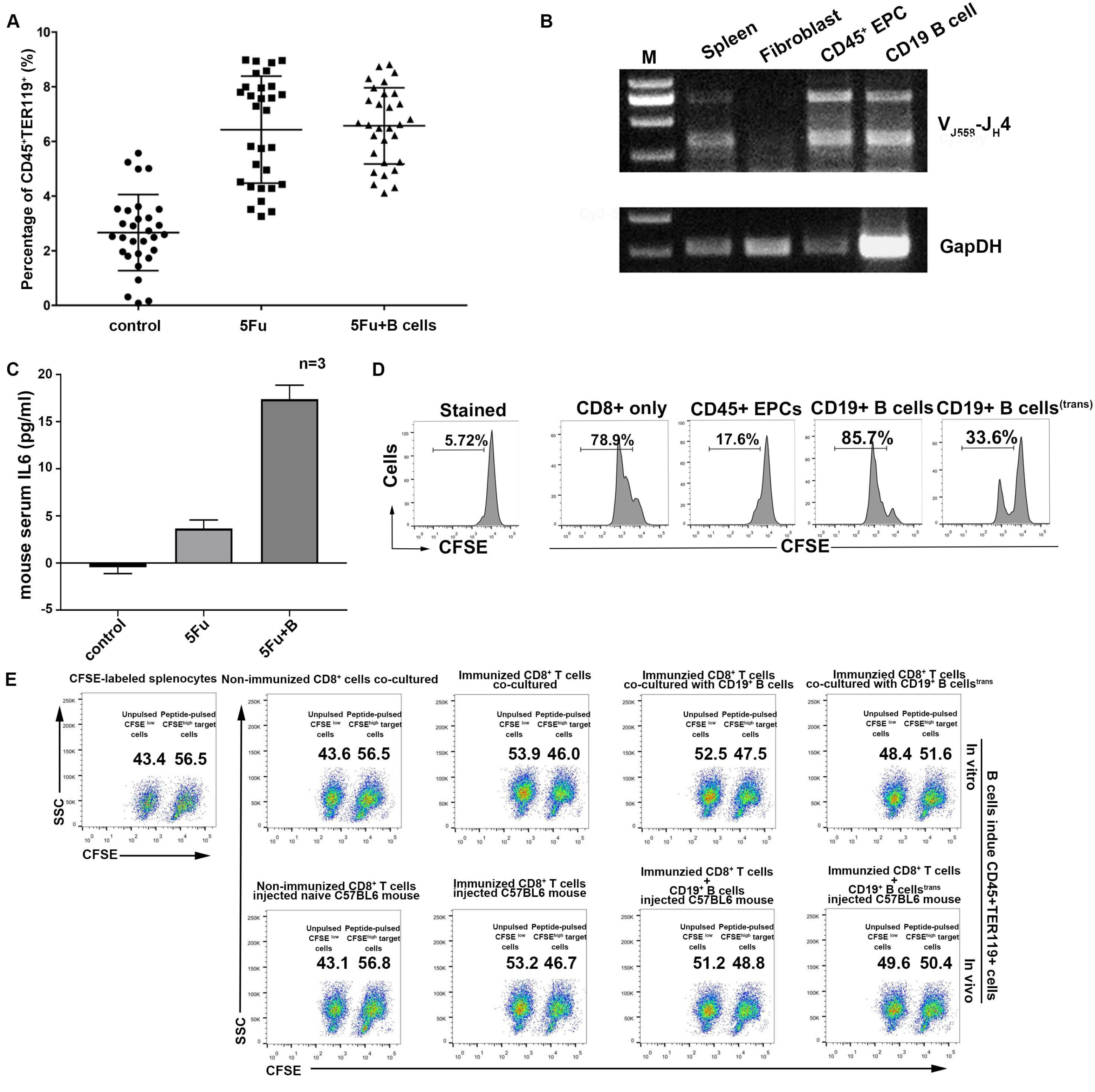

Supplemental Figure 6

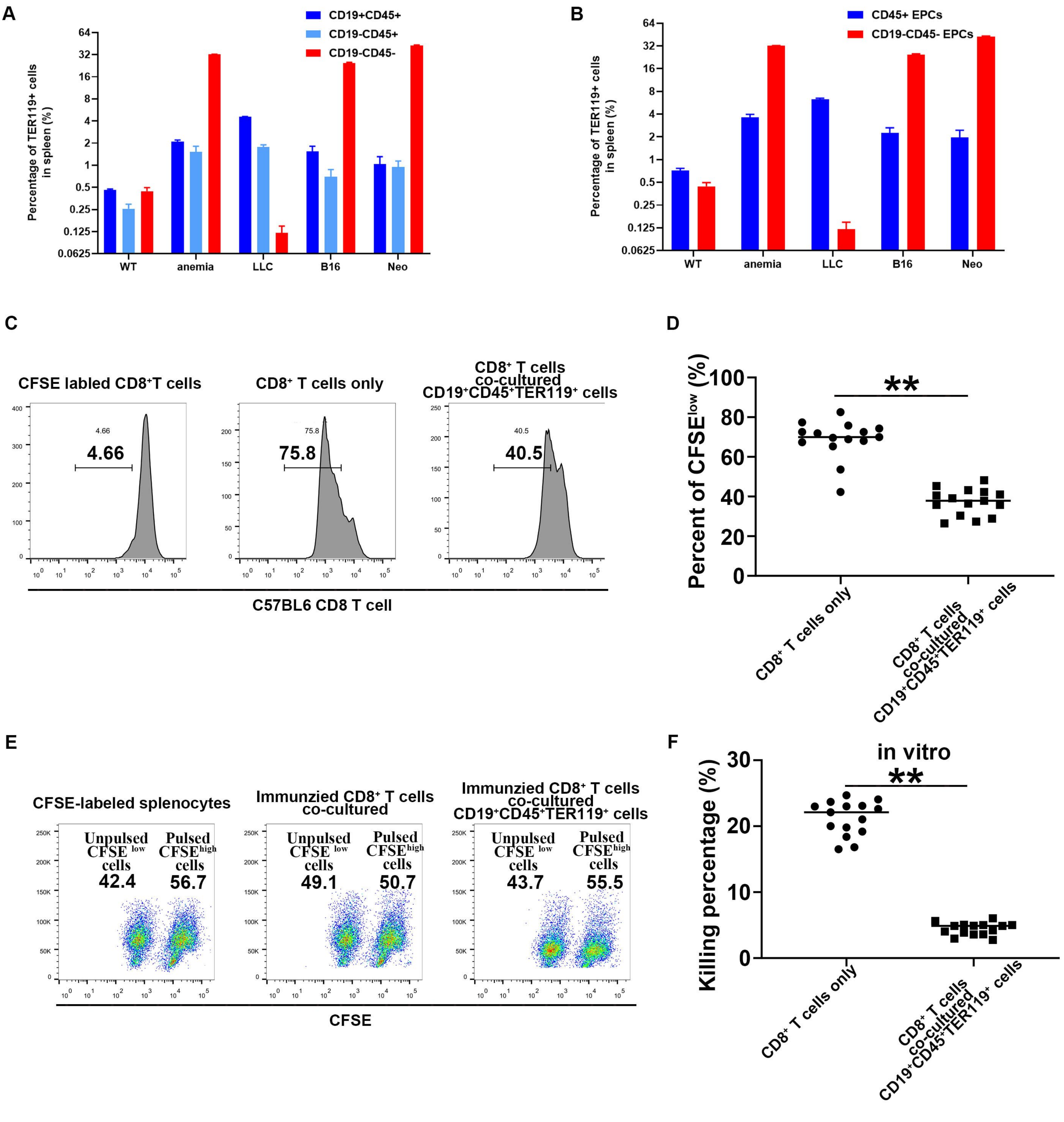

Supplementai Figure 7

BD FACSDiva 8.0.2

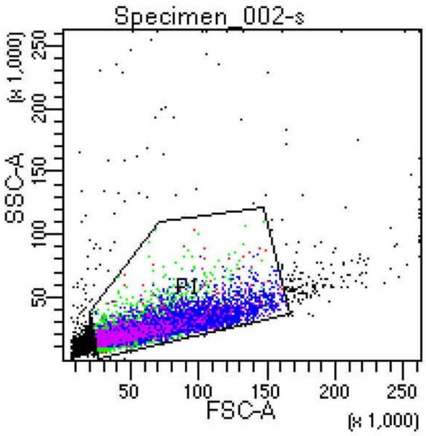

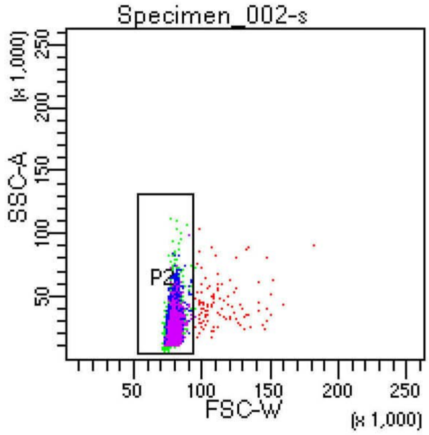

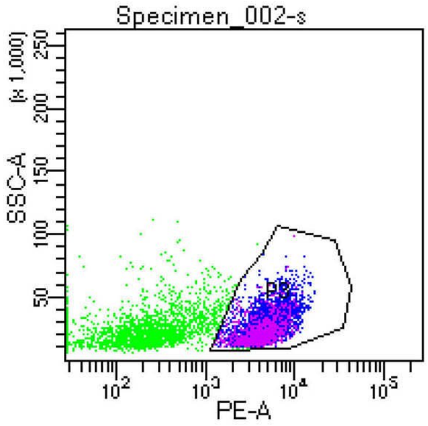

CD19

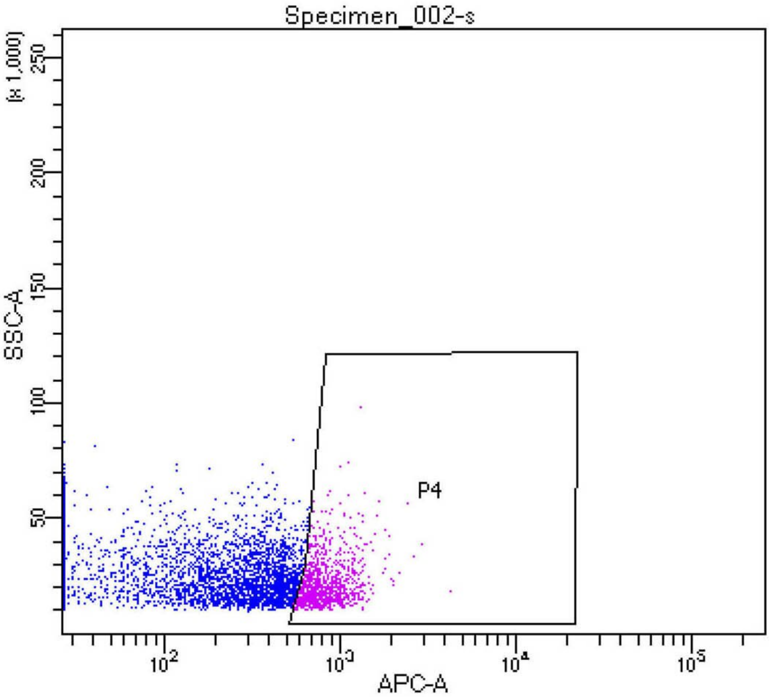

TER119

Neonatal spleen CD19+TER119+ cells

Tube: s

Population	#Events	%Parent	%Total
All Events	10,000	####	100.0
P1	7,876	78.8	78.8
P2	7,761	98.5	77.6
P3	5,550	71.5	55.5
P4	989	17.8	9.9

Supplemental Figure 8

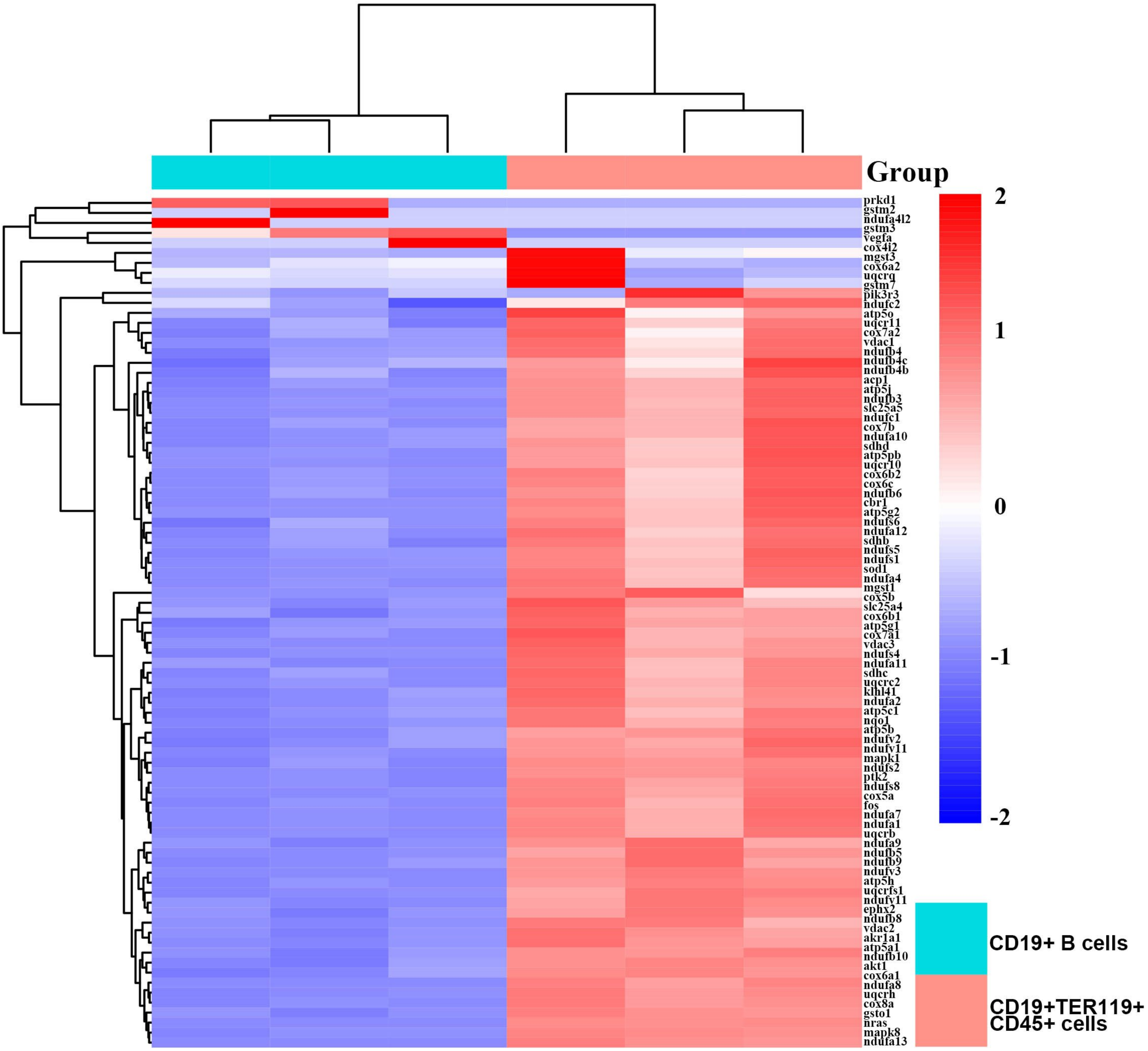

KEGG:Chemical carcinogenesis - reactive oxygen species (mmu05208)

Supplemental Figure 9

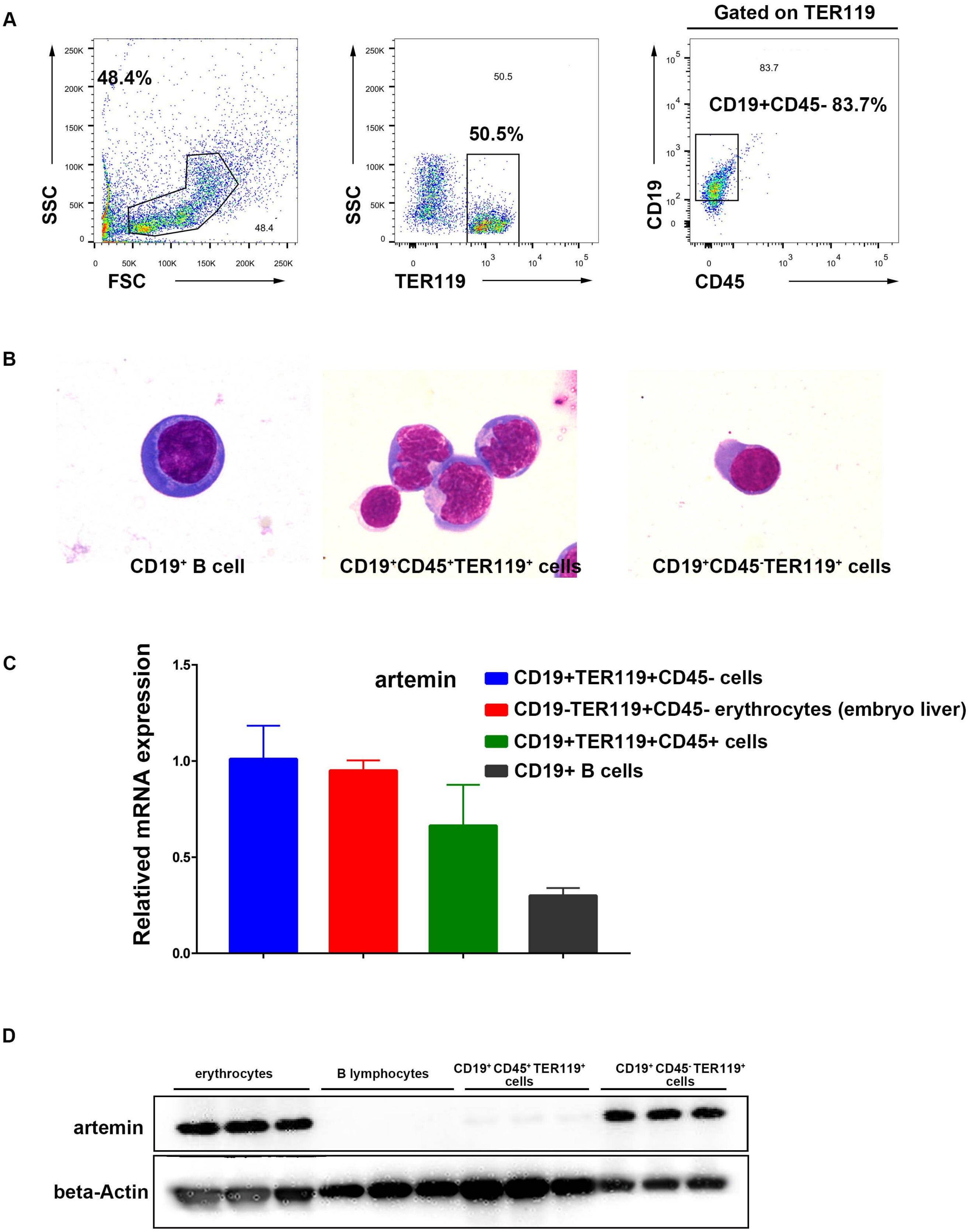

Supplemental Figure 10

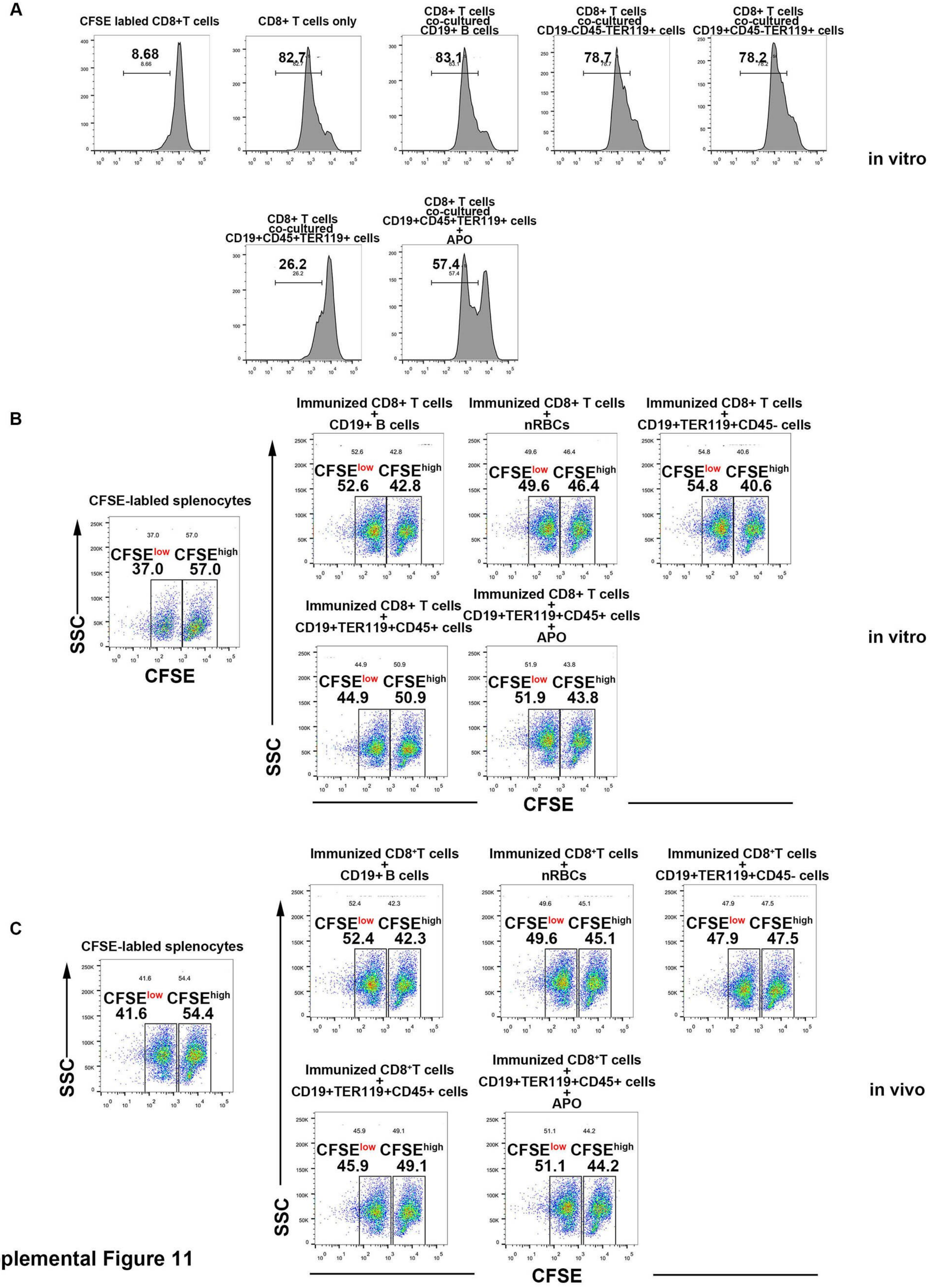

Supplemental Figure 11

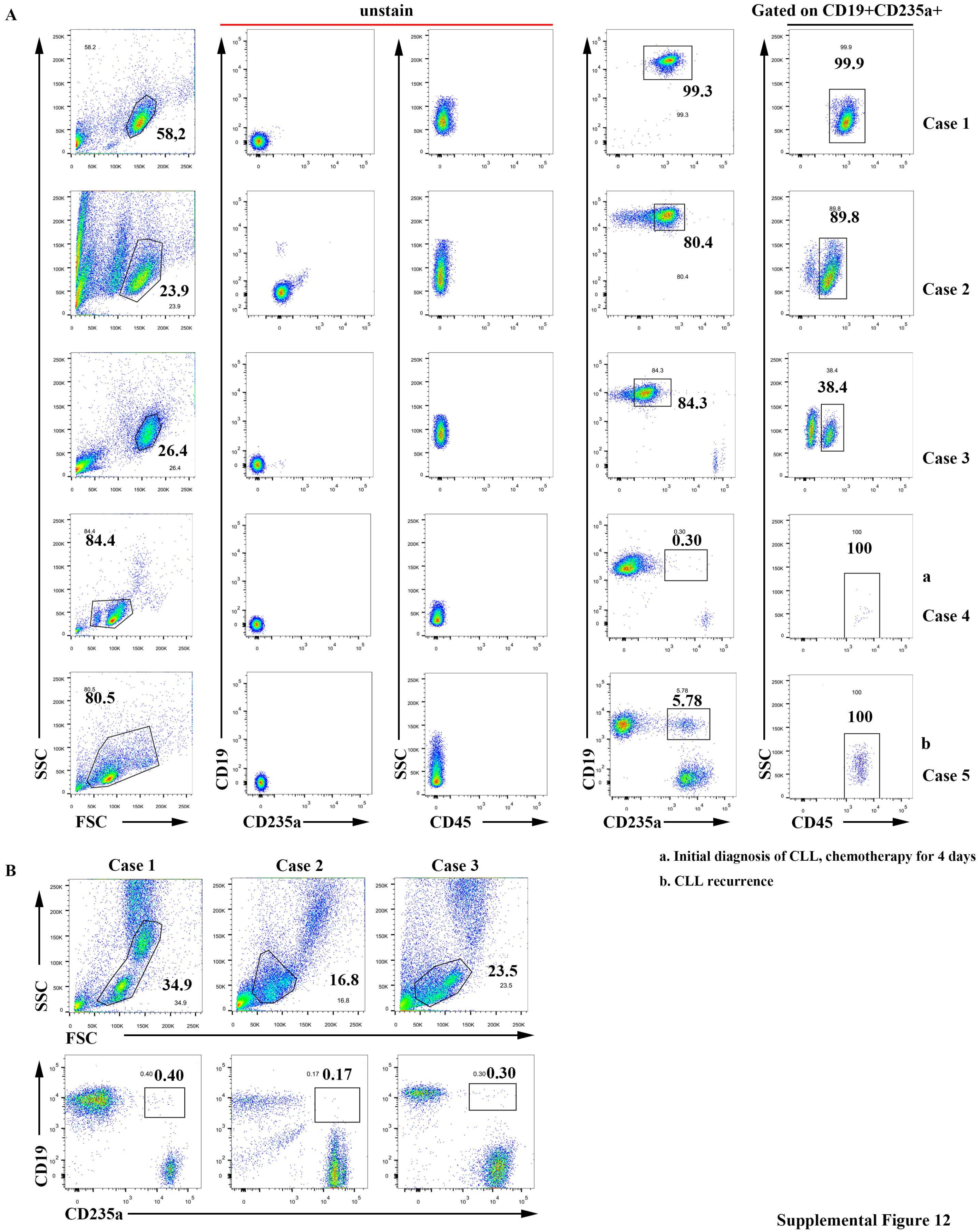

a. Initial diagnosis of CLL, chemotherapy for 4 days

b. CLL recurrence

Supplemental Figure 12

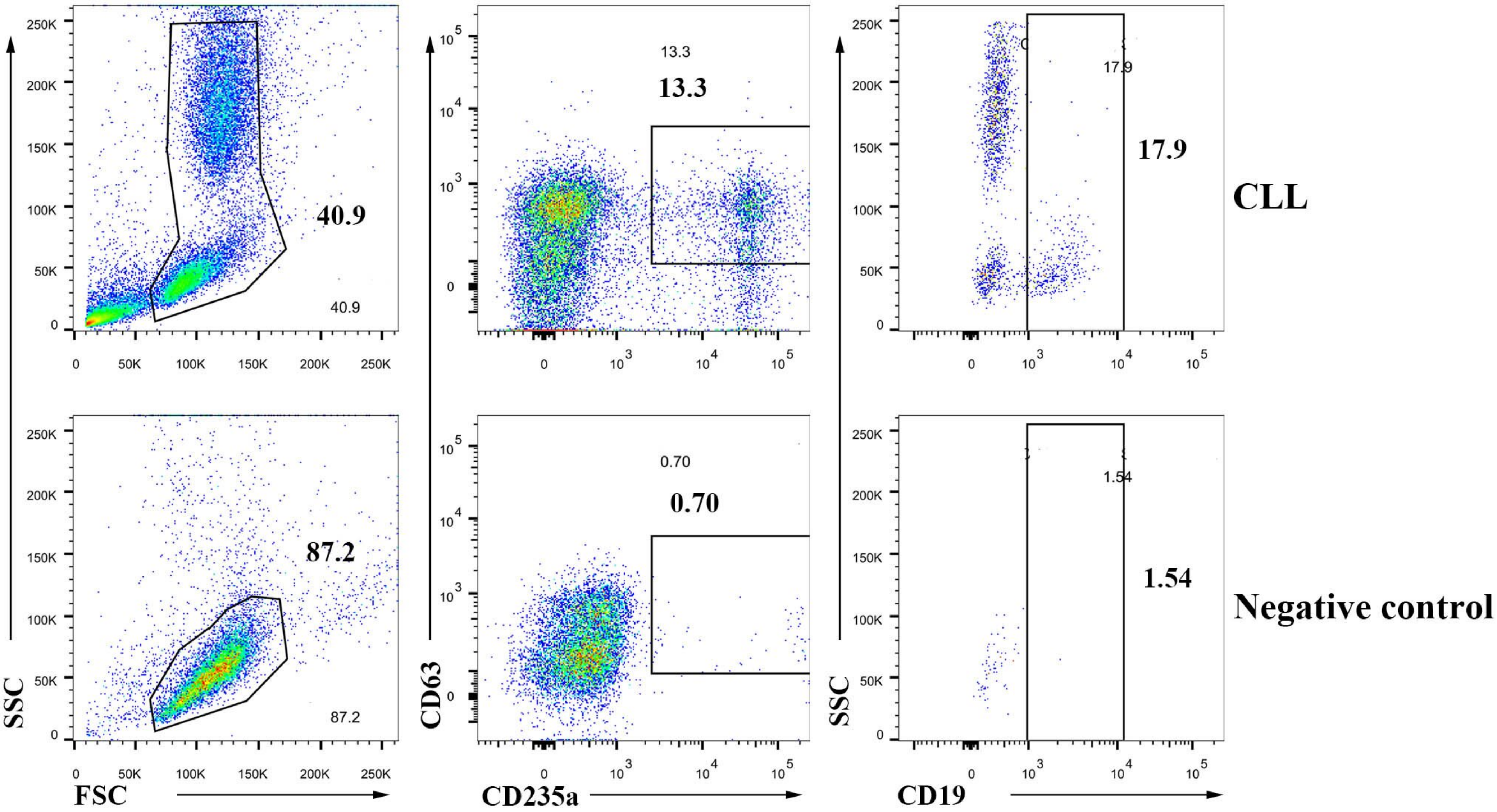

Supplemental Figure 13
