# Supplementary material for: B lymphocytes transdifferentiate into immunosuppressive erythroblast-like cells
Source: Front Immunol. 2023 Jul 21;14:1202943. doi: 10.3389/fimmu.2023.1202943 (PMC10401433; doi:10.3389/fimmu.2023.1202943)
Supplement: Supplementary file 3 [file Table_1.docx]

# Table 1

Primer sequences used for genomic DNA PCR

| Gene | Sequence (5’ to 3’) |
| --- | --- |
| ER1-gag-myc-S | TTGTACACCCTAAGCCTCCG |
| ER1-gag-myc-A | TGCTGCTAGAAGTTCTC |
| V_J558_-J_H_4-S | CAGGTCCAACTGCAGCAG |
| V_J558_-J_H_4-A | CTCTCAGCCGGCTCCCTCAGGG |
| FR3A | ACACGGCYSTGTATTACTGT |
| LJH | TGAGGAGACGGTGACC |
| VLJH | GTGACCAGGGTNCCTTGGCCCCAG |

S: sense primer; A: anti-sense primer

Primer sequences used for qRT-PCR

| Gene | Sequence (5’ to 3’) |
| --- | --- |
| Mus_GAPDH-S | GTCCCTCACCCTCCCAAAAG |
| Mus_GAPDH-A | GCTGCCTCAACACCTCAACCC |
| Mus_Actin-S | GAGACCTTCAACACCCCAGC |
| Mus_Actin-A | ATGTCACGCACGATTTCCC |
| Mus_EBF1-S | ACCCTGAAATGTGCCGAGTA |
| Mus_EBF1-A | GGAGTCTCATTTCGGTTGCC |
| Mus_Pax5-S | GCAGATTAGCCAACCCACAG |
| Mus_Pax5-A | AGGCACTGCCAGTTTCAAAG |
| Mus_Nox2-S | TCCTATGTTCCTGTACCTTTGTG |
| Mus_Nox2-A | GTCCCACCTCCATCTTGAATC |
| Mus_Arg2-S | GATCTCTGTGTCATCTGGGTTG |
| Mus_Arg2-A | AATCCTGGCAGTTGTGGTAC |
| Mus_CD22-S | CGTCTGGGTCATGGAAAGATAG |
| Mus_CD22-A | TCTGGATTGCTGGAGTTGTAC |
| Mus_CD19-S | GGAAAAGGAAGCGAATGACTG |
| Mus_CD19-A | AGAGGTAGATGTAGGAAGGGAG |
| Mus_CD79a-S | GTGAAAACAATGGCAGGAACC |
| Mus_CD79a-A | CTGTGGTTCTTGTTTACTTCGG |
| Mus_CD79b-S | ACCCTCACTATCCAAAACATCC |
| Mus_CD79b-A | TCCAACGTGCTGAATCCTAAG |
| Mus_CD20-S | AAAACTCCCCATCTACACAGTAC |
| Mus_CD20-A | CCACTCATTCTCCACAATACCAG |
| Mus_Ptprc-S | GACAGAGTTAGTGAATGGAGACC |
| Mus_Ptprc-A | AAAAGTTCGGAGAGTGTAGGC |
| Mus_Gata1-S | TGGTGTCCTCACCATCAGAT |
| Mus_Gata1-A | CTCAGCTTCTCTGTAGTAGG |
| Mus_Uros-S | CTAGAAGCCACACTGATTCC |
| Mus_Uros-A | GTCCTTCTCCAAACACAGCT |
| Mus_Hba-a1-S | AAGCAACATCAAGGCTGCCT |
| Mus_Hba-a1-A | CCGTGGCTTACATCAAAGTG |
| Mus_Hbb-b1-S | ATGCTGAGAAGGCTGCTGTC |
| Mus_Hbb-b1-A | TGATAGCAGAGGCAGAGGAT |
| Mus_Ppox-S | CAGAGTGACTGTGATGTTGG |
| Mus_Ppox-A | GCAGTGACTCGGTGGTTCTT |
| Mus_Klf1-S | CTCCATCAGTACACTCACCA |
| Mus_Klf1-A | TTCAGACTCACGTGATGGGA |
| Mus_Urod-S | AGCTGAAGAATGACACGTTC |
| Mus_Urod-A | GCAGGTGCTGAAGAAGTCCT |
| Mus_Ank1-S | AGTCCTTCTGCAGAATGACC |
| Mus_Ank1-A | TGAGGTGTGAAGTTGACGCT |
| Mus_Gypa-S | AACCACTCCTGTGGTGGCTT |
| Mus_Gypa-A | TGTGTGGTGAGACAGGCTGT |
| Mus_Hmbs-S | TGGACCTAGTGAGTGTGTTG |
| Mus_Hmbs-A | CTCCACCAGTCAGGTACAGT |
| Mus_Slc4a1-S | TGACCTTCTGGAGCCTTCTA |
| Mus_Slc4a1-A | CTGATCCTCGTAGATGAAGC |
| Mus_Alas2-S | GTGGAGCTTGAACAGGAGCT |
| Mus_Alas2-A | CCTGCATCTGAGTAGATCTC |
| Mus_Stom-S | CTCATCAAGGTGGACATGAG |
| Mus_Stom-A | GCTGAATCTGCATTGGTGAT |
| Mus_Nfe2-S | CTCAACTACAGTGATGCAGA |
| Mus_Nfe2-A | TGTCTCAGTGGGTGGAAGAG |
| Mus_Ebp4.9-S | ATCGAGGACCTCATCATCGA |
| Mus_Ebp4.9-A | CGAGATGCCTTCCGTTTCCT |
| Mus_Myb-S | GACACAGCATCTACAGTAGC |
| Mus_Myb-A | CACCAGCTTCTTCAGCTTCT |
| Mus_Gata2-S | TTCAACCATCTCGACTCGCA |
| Mus_Gata2-A | GCTGTGCAACAAGTGTGGTC |
| Mus_Tal1-S | ACTAGGCAGTGGGTTCTTTG |
| Mus_Tal1-A | CGCACTACTTTGGTGTGAGG |
| Mus_Ldb1-S | GCTGACCATCACTTTCTGCT |
| Mus_Ldb1-A | TTGCTGTGGAATGCCTCCTT |
| Mus_Lmo2-S | GATGAGGTGCTGCAGATACC |
| Mus_Lmo2-A | AGAGGTCACAGCTGAGGCAA |
| Mus_Fech-S | AGATGTGGACTTCCAAGCAA |
| Mus_Fech-A | CTAGTCCATCTCTCTCCATC |
| Mus_EpoR-S | TGAGTGTGTTCTGAGCAACC |
| Mus_EpoR-A | AAGATGAGAGGGTCCAGGTC |
| Mus_Diaph3-S | AGAAGCGACCCAAGTTGCAT |
| Mus_Diaph3-A | GTGATGAGCTGTCACTGATC |
| Mus_Artn-S | GCTATGAGGCCGTCTCCTTC |
| Mus_Artn-A | GGGTCTAGTGTGCAAAGGCT |

S: sense primer; A: anti-sense primer
